# *BRCA1* and *BRCA2* gene expression: p53- and cell cycle-dependent repression requires RB and DREAM

**DOI:** 10.1038/s41418-025-01566-9

**Published:** 2025-08-22

**Authors:** Marianne Quaas, Robin Kohler, Lukas Nöltner, Louisa F. Schmidbauer, Sigrid Uxa, Gerd A. Müller, Kurt Engeland

**Affiliations:** 1 Molecular Oncology, Faculty of Medicine, University of Leipzig, Leipzig, Germany; 2 Present Address: Department of Clinic of Visceral, Transplantation, Thoracic, and Vascular Surgery, University Hospital Leipzig, Faculty of Medicine, University of Leipzig, Leipzig, Germany; 3 Present Address: Department of Hematology, Cellular Therapy, Hemostaseology and Infectious Diseases, University Hospital Leipzig, Faculty of Medicine, University of Leipzig, Leipzig, Germany; 4 Present Address: Department of Chemistry and Biochemistry, University of California, Santa Cruz, CA USA

**Keywords:** Cancer genetics, Gene expression, Cell biology

## Abstract

BRCA1 and BRCA2 proteins are crucial for DNA repair through homologous recombination (HR), which predominantly takes place during S and G_2_ phases. Their expression is tightly regulated to ensure HR occurs exclusively within these phases. While these proteins are well-established tumor suppressors in hereditary breast and ovarian cancers, their inactivation is rare across all sporadic cancers. Counterintuitively, BRCA1 and BRCA2 expression is downregulated rather than upregulated following DNA damage and p53 activation. In this study, we demonstrate that *BRCA1* and *BRCA2* gene expression is governed by the same transcriptional mechanisms throughout the cell cycle, peaking in the S phase. During G_0_/G_1_ and following p53 activation, *BRCA1/2* transcription is repressed by DREAM and RB:E2F repressor complexes. Importantly, this transcriptional repression occurs concurrently with the coordinated downregulation of numerous genes involved in cell cycle control and DNA repair pathways. Consistent with previous findings, this suppression notably affects members of the Fanconi anemia group and is mediated through the DREAM complex. Such broad transcriptional suppression facilitates exit from S phase, thereby promoting a fundamental shift in DNA repair mechanisms. Following DNA damage, we demonstrate that *BRCA1/2* downregulation occurs indirectly through the p53-p21-DREAM/RB axis, wherein p53-induced *p21/CDKN1A* expression initiates repression dependent on DREAM and RB. These results, together with observations from previous studies, suggest that DNA repair shifts from HR to the error-prone pathways of non-homologous end joining (NHEJ) and single-strand annealing (SSA), resulting in chromosomal aberrations and cell death, thereby in fact preventing malignant transformation. Our findings elucidate the transcriptional regulation of *BRCA1* and *BRCA2* expression. These regulatory mechanisms, when considered alongside prior findings and hypotheses, may help explain why BRCA1 and BRCA2 proteins do not exhibit tumor-suppressive functions in most cell types.

## Introduction

*BRCA1* and *BRCA2* were discovered as tumor suppressor genes in hereditary breast and ovarian cancer [1, 2]. However, autosomal dominant mutations in these genes also predispose individuals to pancreatic, stomach, laryngeal, fallopian tube, and prostate cancer. The highest lifetime risks of developing malignant disease due to inherited *BRCA1* and *BRCA2* mutations are observed in breast cancer, with rates of 70–80% and 50–60%, respectively. The risks for ovarian tumors are 50% and 30%, respectively [3]. In contrast to germline mutations, sporadic mutations in *BRCA1* or *BRCA2* are rarely observed [4, 5]. Consequently, it has been suggested that there are fundamental differences between early-onset cancers carrying germline mutations and late-developing tumors with somatic mutations [4]. Additionally, indicating a role in development, inactivating homozygous mutations of *Brca1* and *Brca2* lead to embryonic lethality in mice [6].

BRCA1 and BRCA2 are expressed in all tissues and play essential roles in DNA repair [3, 7, 8]. Given their function in genomic integrity, they could be expected to act as tumor suppressors across all tissues. However, this is not observed in most tissue types or non-hereditary tumors with *BRCA1/BRCA2* mutations [4, 5, 9]. Surprisingly, young breast cancer patients with these mutations show no overall survival disadvantage. A large prospective study comparing early-onset breast cancer patients with *BRCA1/BRCA2* germline mutations to those with sporadic cancer found no significant survival difference. Only triple-negative breast cancer (TNBC) patients showed a slight disparity within two years of diagnosis. Remarkably, *BRCA1/BRCA2* mutation carriers even had a small but significant survival advantage over non-carriers [10]. Thus, BRCA1/BRCA2’s strong tumor-suppressive effects appear largely confined to hereditary breast and ovarian cancer.

Clinical studies indicate that BRCA1/BRCA2 loss can enhance therapy efficacy by increasing cancer cell sensitivity to DNA-damaging treatments [11–13]. In TNBC, reduced BRCA1/BRCA2 expression correlates with better chemotherapy response, linked to the high mutational burden from defective DNA repair [11]. Ovarian cancer with BRCA1/BRCA2 inactivation similarly shows heightened sensitivity to DNA-damaging therapy [13].

To fulfill their cellular roles in repair, BRCA1 and BRCA2 proteins functionally and physically interact to regulate common pathways involved in the DNA damage response (DDR) and checkpoint control of the cell cycle [3, 9, 12, 14–18]. These proteins link the sensing of DNA damage with the initiation and catalysis of DNA repair. Counterintuitively, and despite their well-established role in DNA repair, *BRCA1* and *BRCA2* expression is downregulated following DNA damage or p53 activation [19–21].

BRCA1 and BRCA2 play key roles in the repair of double-strand breaks (DSBs) by homologous recombination (HR) [22]. In general, the type of DNA repair depends on the phase of the cell cycle. HR is the predominant repair mechanism during S and G_2_ phases, whereas non-homologous end joining (NHEJ) is employed in quiescence and G_1_ phase.

Thus, BRCA1/2 expression needs to be controlled during the cell cycle. Generally, cell cycle-dependent transcription of numerous genes is regulated by either the RB:E2F or DREAM/MuvB complexes. In the RB:E2F system, the retinoblastoma tumor suppressor RB (*RB1*) functions as a repressor of E2F transcription factors, which bind to E2F sites in the promoters of target genes. This RB:E2F repressor complex downregulates gene expression in G_0_ and early G_1_. A shift in RB phosphorylation status later in the cell cycle leads to the dissociation of RB from E2F, enabling gene activation by E2F transcription factors through E2F sites [23–25].

The DREAM transcriptional repressor complex downregulates target genes in resting and early G_1_ cells binding to E2F or CHR (*cell cycle genes homology region)* sites [26, 27]. With binding to the CHR site the repertoire of DREAM target genes is broader than that of RB:E2F complexes [28]. Following phosphorylation-dependent dissociation of its RB-like components, DREAM switches its composition to form the MuvB core complex, which subsequently associates with the oncogenes A-MYB, B-MYB, and FOXM1 [26, 28–33]. Thus, the transition from DREAM to A-MYB:MuvB, B-MYB:MuvB, and FOXM1:MuvB complexes drives a shift from repression to activation of the same target genes [29, 34].

One key property of the RB:E2F and DREAM complexes is their role as transcriptional repressors in the indirect regulation mediated by the tumor suppressor p53. Upon activation, p53 directly transactivates the *p21/CDKN1A* gene. The CDK inhibitor p21 then prevents hyperphosphorylation of RB and RB-like proteins, stabilizing the RB:E2F and DREAM complexes [21, 35, 36]. This results in the downregulation of hundreds of genes through the p53-p21-RB/DREAM pathways [25, 34]. Ultimately, indirect transcriptional repression by p53 via these pathways leads to cell cycle arrest [37].

Understanding the regulation of *BRCA1* and *BRCA2* expression is crucial for elucidating how their encoded proteins are connected to signaling pathways that link *BRCA1/2* expression to cell cycle control, DNA repair, and the response to p53 activation. Although several studies have addressed aspects of the transcriptional regulation of *BRCA1* and *BRCA2*, the available information remains fragmented. For instance, early reports identified binding sites for transcription factors such as E2F1 and E2F4, as well as RB-related proteins p107 (RBL1) and p130 (RBL2), in the upstream regulatory region of the *BRCA1* gene, yet failed to detect binding by RB itself [38]. Another study demonstrated cell cycle-dependent expression of *BRCA2* mRNA and provided evidence for a functional E2F-binding site within its promoter. However, despite extensive electrophoretic mobility shift assays (EMSAs) investigating multiple E2F proteins, no binding of RB:E2F complexes was detected [39]. Additionally, Cyclin D1/CDK4-dependent activation of *BRCA1* transcription has been observed [40]. More recently, genome- and transcriptome-wide analyses have suggested that both the DREAM and RB:E2F complexes contribute to the transcriptional regulation of *BRCA1* and *BRCA2*. For instance, RB binding has been detected in the promoter regions of both genes [41], and DREAM complex components — E2F4, p130, LIN9, and LIN54 — have also been shown to bind these promoters [21]. In a more detailed study, E2F4 was found to bind the promoters of *BRCA1*, *BRCA2*, and several other Fanconi anemia genes following p53 induction, implicating DREAM-mediated repression in this context [20]. Despite these advances, key aspects of the regulatory landscape remain unclear. These include the potential involvement of other transcription factors — such as activating E2Fs, A-MYB, B-MYB, and FOXM1 — in regulating *BRCA1* and *BRCA2* expression, the precise locations of promoter binding sites, and the possible co-regulatory roles of the RB:E2F and DREAM/MuvB complexes.

In this study, we investigate the mechanisms regulating the cell cycle-dependent transcription of *BRCA1* and *BRCA2*, as well as their response to p53 activation. Our findings suggest that the regulatory mechanisms controlling *BRCA1* and *BRCA2* expression are highly similar. We elucidate how *BRCA1* and *BRCA2* expression is integrated into signaling pathways that regulate cell cycle progression and DNA repair. Additionally, our findings provide mechanistic insight that supports and extends a previously proposed rationale for why BRCA1 and BRCA2 typically fail to exert tumor-suppressive functions in most cell types.

## Materials and methods

### Sequence analyses

Potential regulatory, evolutionary conserved elements in the *BRCA1* and *BRCA2* genes were identified with the UCSC genome browser by comparing promoter sequences of six mammalian species and by applying the 100 vertebrate conservation track [42].

### Cell culture and drug treatment

RPE-1, NIH3T3, HFF, and T98G cells (DSMZ, Braunschweig, Germany) as well as HCT116 wild-type, *p53*^*-/-*^ and *p21*^*-/-*^ cells [43] were grown in DMEM (Lonza) supplemented with 10% FCS and penicillin/streptomycin and maintained at 37 °C and 10% CO_2_. RPE-1, NIH3T3, HFF, and T98G cells were synchronized in G_0_ by serum starvation (0% FCS) for 60–72 h or density-arrest. For cell cycle analyses, cells were stimulated to re-enter the cell cycle with 20% FCS after serum deprivation. HCT116 cells were treated with 0.2 µg*/*ml doxorubicin (Medac) or 10 µM nutlin-3a (Cayman Chemicals) for 24 h to 48 h. Control cells were treated with the solvent DMSO or left untreated.

### Knockout cell lines

HCT116 wild type (WT), HCT116 *p53*^*−/−*^, and HCT116 *p21*^*−/−*^ cells were a generous gift from Bert Vogelstein [43]. *LIN37*^*-/-*^ and *RB*^*-/-*^ knockouts of NIH3T3 and HCT116 cell lines as well as double-knockouts (DKO) were generated via a CRISPR/Cas9 nickase approach [37, 44].

### RNA extraction, reverse transcription, and semi-quantitative real-time PCR

Total RNA was isolated using TRIzol Reagent (Invitrogen) following the manufacturer’s protocol. One-step reverse transcription and quantitative real-time PCR were performed with an ABI 7300 system (Applied Biosystems) using the QuantiTect SYBR Green PCR kit (Qiagen) or the GoTaq® 1-Step RT-qPCR System (Promega). *U6* served as an endogenous control. Sequences of primers can be obtained upon request.

### Plasmids

Promoters of human *BRCA2* (nt −320 to +196, relative to the transcription start site) and *BRCA1* (nt −505 to −1, relative to the translation start) were amplified from genomic DNA extracted from HFF cells by standard PCR. DNA fragments were cloned into the pGL4.10 luciferase reporter vector (Promega). Site-directed mutagenesis was performed following the QuikChange protocol (Agilent Technologies). Primer sequences used for cloning and creating mutations can be obtained upon request. The expression plasmids for human p53, pcDNA-p53wt, and pcDNA-p53mut (R175H) were created by amplifying the insert of pcDNA3.1HisC-p53 [36] and ligating it into pcDNA3.1(+)3x-Flag (C-terminal). Expression plasmids for human p21/CDKN1A^WAF1/CIP1^, pcDNA-p21wt, and pcDNA-p21mut were cloned by amplifying the inserts of pCEP-p21wt and pCEP-p21mut, respectively [45], and ligation in pcDNA3.1(+).

### DNA affinity purification

DNA affinity purifications were performed as described earlier [46]. Proteins binding to biotinylated promoter probes were purified from nuclear extracts of density-arrested NIH3T3 cells, restimulated RPE-1 cells, or proliferating HCT116 cells, and detected by Western blot. As a positive control for E2F promoters, a *Dhfr* promoter probe and as negative controls, a fragment of the mouse *Gapdhs* promoter or the mouse *Cyclin B2* promoter CHR mutant probe was employed [26].

### Chromatin immunoprecipitation (ChIP)

ChIPs and quantification of promoter fragments by semi-quantitative real-time PCR were performed as described previously [26, 36]. The following antibodies were employed for immunoprecipitation: E2F4 (C-20, Santa Cruz Biotech. and E3G2G, Cell Signaling Technology), p130 (C-20, Santa Cruz Biotech. and D9T7M, Cell Signaling Technology), E2F1 (C-20, Santa Cruz Biotech.), E2F3 (C-18, Santa Cruz Biotech.), B-MYB (N-19, Santa Cruz Biotech. and A301-655A, Bethyl), A-MYB (HPA008791, Sigma-Aldrich), FoxM1 (D3F2B, Cell Signaling Technologies), LIN9 (ab62329, Abcam), LIN37 [31, 44], and p53 (Ab-6, DO-1, Calbiochem). Primer sequences can be obtained upon request. Protein binding to the *GAPDHS* promoter served as a negative control.

### SDS-PAGE and Western blot

SDS-PAGE and Western blot were performed following standard protocols [47]. The following antibodies were applied for protein detection: E2F4 (C-20, sc-866; Santa Cruz Biotech.), p130 (RBL2, D9T7M; Cell Signaling Technology), LIN9 (A300-BL2981, Bethyl Laboratories), LIN37 (T3, custom-made at Pineda Antikörper-Service, Berlin, Germany, [28]), BRCA1 (D-9, sc-6954; Santa Cruz Biotech.), BRCA2 (A303-434A, Bethyl), B-MYB (provided by Roger Watson), Survivin (71G4B7, Cell Signal Technology), Ki-67 [48], Kif23 (MKLP-1, sc-136473 #24, Santa Cruz Biotech.), MCM5 (sc-136366 #33, Santa Cruz Biotech.), Cyclin E1 (E-4, Santa Cruz Biotech.), E2F1 (A300-766A, Bethyl), RB (D20, No. 9313; Cell Signaling Technologies), p21 (Ab-1, EA10; Merck/Calbiochem), β-actin (A5441, Sigma-Aldrich) and LIN54 (A303-799A, Bethyl Laboratories). Original blot data are provided as Suppl. Fig. Uncropped Westerns.

### Transfections and luciferase promoter reporter assays

Cell cycle-dependent promoter activities were analyzed by luciferase reporter assays with extracts of transfected serum-starved and restimulated NIH3T3 cells as described before [26]. In order to measure p53-dependent promoter activity, HCT116 *p53*^*-/-*^ and *p21*^*-/-*^ cells were plated in 24-well plates (75,000 cells per well) and transfected by GeneJuice (EMD Millipore) with 100 ng of promoter reporter plasmids (pGL4.10) along with 50 ng of constructs expressing wild-type or mutant p53 or p21 proteins [36]. After 24 h, cells were collected and luciferase activity was measured with the Dual*-*Luciferase Reporter Assay system (Promega). Transfection with siRNAs was performed with HCT116 cells by reverse transfection of 1 × 10^6^ cells in a 5 cm dish and 5 ml growth medium with 20 nM total siRNA and 5 μl Lipofectamine™ RNAiMAX (Thermo Fisher).

### Flow cytometry

For DNA content analysis, cells were fixed in one volume PBS/1 mM EDTA and three volumes of absolute ethanol overnight at 4 °C, centrifuged for 8 min at 500 x *g*, and resuspended in PBS/1 mM EDTA. DNA was stained with propidium iodide at a final concentration of 20 μg/ml. The DNA content of the cells was analyzed by staining with propidium iodide (PI) followed by flow cytometry [49, 50].

### Software

Data were analyzed using GraphPad Prism10.2 (GraphPad Software, Boston, MA).

## Results

### BRCA1 and BRCA2 mRNA and proteins exhibit maximal expression during the S phase of the cell cycle

**Fig. 1:**
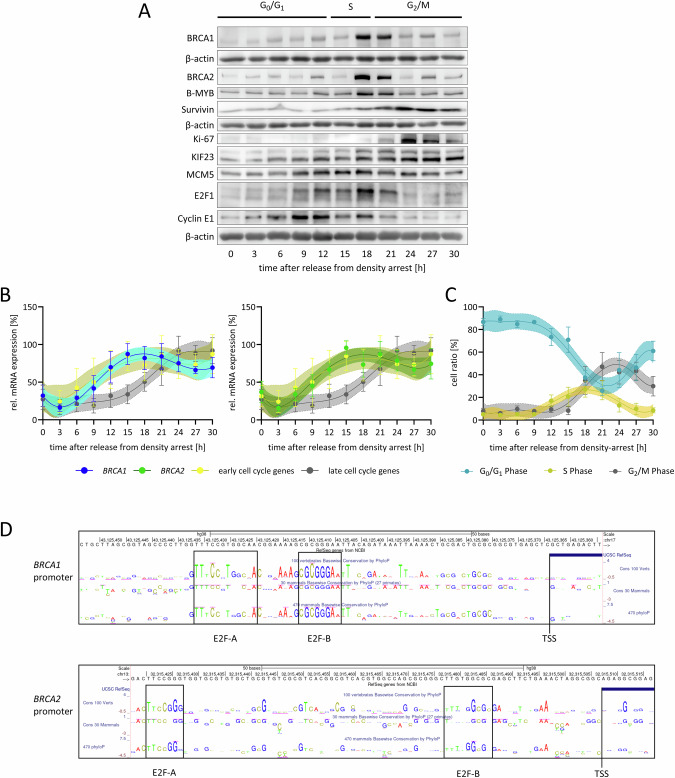
BRCA1 and BRCA2 mRNA and proteins exhibit maximal expression during the S phase of the cell cycle. **A** RPE-1 cells were arrested (0 h time point) and subsequently stimulated to enter the cell cycle. BRCA1 and BRCA2 protein expression was analyzed by Western blot and compared to the early cell cycle proteins B-MYB, Cyclin E1, E2F1, and MCM5 as well as the late cell cycle proteins KIF23, Ki-67, and Survivin (representative replicate from *n* = 4). Four lines with β-actin detection served as loading control for the four individual gels. **B** *BRCA1* and *BRCA2* mRNA expression normalized to U6 RNA expression was measured in density-arrested and released RPE-1 cells and compared to a set of early cell cycle genes (*CCNE1*, *DHFR*, *ORC1*) as well as a set of late cell cycle genes (*Survivin/BIRC5*, *CCNB2*, *CDC25C*). Mean ± SD are given and sixth order polynomial regressions with 95% CI were calculated (*n* = 4). **C** As an analysis for cell cycle distribution in (**B**), DNA content staining was analyzed by flow cytometry and cells were grouped into G_0_/G_1_, S, or G_2_/M phase based on their DNA content. Mean ± SD are given and sixth order polynomial regressions with 95% CI were calculated (*n* = 4). **D** Evolutionarily conserved and potentially regulatory elements in the human *BRCA1* and *BRCA2* promoters were identified using the *UCSC Genome Browser*. This was achieved by comparing sequences from seven mammalian species and employing the 100 vertebrates conservation track. The analysis spans 100 nucleotide regions, from −90 to +10 relative to the transcription start site (TSS), revealing potential transcription factor binding sites of biological significance.

The expression of BRCA1 and BRCA2 was analyzed throughout the cell cycle (Fig. 1). RPE-1 cells were density-arrested in G_0_ and subsequently released to progress through the cell cycle. BRCA1 and BRCA2 protein levels peaked during S phase and extended to some extent into G_2_ phase, while their expression remained low or at background levels during other cell cycle phases, including G_0_. (Fig. 1). For reference, B-MYB, Cyclin E1, E2F1, and MCM5 were analyzed as examples of early cell cycle proteins, while KIF23, Ki-67, and Survivin were assessed as markers of late cell cycle phases.

Similarly, *BRCA1* and *BRCA2* mRNA expression displayed a pattern consistent with the protein levels, with mRNA peak levels observed in S phase, preceding the protein peak by approximately one to two hours (Fig. 1). This S phase-specific expression pattern for *BRCA1* and *BRCA2* was also confirmed in other cell systems, including human foreskin HFF fibroblasts and mouse NIH3T3 cells (Fig. S1).

To explore the mechanisms underlying the cell cycle-dependent expression pattern, we examined the promoter regions of the two genes for phylogenetically conserved nucleotide sequences. We identified two conserved sites within the promoters of each gene containing consensus binding motifs for E2F transcription factors (Fig. 1D).

### *Brca1* and *Brca2* downregulation in G_0_ depends on Lin37/DREAM and Rb

**Fig. 2:**
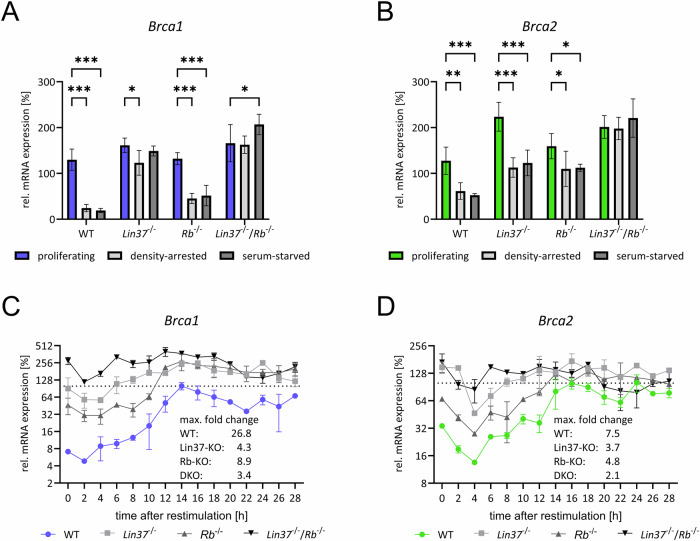
*Brca1* and *Brca2* mRNA downregulation in G_0_ depends on Lin37/DREAM and Rb. Wild-type (WT), *Lin37*^*-/-*^ (Lin37-KO), *Rb*^*-/-*^ (Rb-KO) or *Rb*^*-/-*^*/Lin37*^*-/-*^ (DKO) NIH3T3 cells were used. *Brca1* (**A**) and *Brca2* (**B**) mRNA expression was measured from proliferating, density-arrested, or serum-starved cells. **A**, **B** Mean ± SD; n = 4, two-way ANOVA; *p ≤ 0.05; **p ≤ 0.01; ***p ≤ 0.001. Analogous experiments were performed with serum-starved cells predominantly in G_0_ and cells serum-restimulated to re-enter the cell cycle. From G_0_ and restimulated cells, mRNA levels of *Brca1* (**C**) and *Brca2* (**D**) were analyzed. Maximal fold-changes were calculated as the ratio of the highest to lowest expression levels. Averages from two technical replicates each from four independent cell lines for each cell variant are shown. Expression was normalized to WT proliferating (**A**,** B**) or to maximum expression of WT (**C**,** D**).

E2F promoter sites are capable of binding both RB:E2F complexes and the DREAM complex [28]. To assess the contribution of these complexes in functional assays, we analyzed *Brca1* and *Brca2* expression in synchronized *Rb*^*-/-*^, *Lin37*^*-/-*^, and *Lin37*^*-/-*^*;Rb*^*-/-*^ (DKO) NIH3T3 cells (Fig. 2). Our previous studies demonstrated that the genetic loss of the DREAM subunit Lin37 disrupts DREAM repressor function while leaving the assembly of the remaining complex intact and without affecting transcriptional activation by MuvB-based activator complexes [44].

In wild-type cells, *Brca1* and *Brca2* mRNA expression was high in proliferating cells and significantly downregulated in G_0_-arrested cells (Fig. 2A, B). However, in *Lin37*^*-/-*^ cells, *Brca1* expression was fully deregulated in arrested cells, reaching levels similar to those in proliferating cells (Fig. 2A). In contrast, *Rb*^*-/-*^ cells showed only mild deregulation. Notably, the *Lin37*^*-/-*^*;Rb*^*-/-*^ DKO cells resemble the expression pattern of *Brca1* observed in *Lin37*^*-/-*^ single-knockout cells. Similarly, *Brca2* expression was partially deregulated in G_0_-arrested *Lin37*^*-/-*^ and *Rb*^*-/-*^ cells (Fig. 2B). However, in DKO cells, *Brca2* downregulation was completely lost in G_0_-arrested cells.

Further analysis during cell cycle progression revealed that *Brca1* and *Brca2* expression peaked during S phase in wild-type cells, with maximal changes of 26.8-fold and 7.5-fold, respectively, from the lowest to the highest expression levels (Fig. 2C, D). Loss of Lin37/DREAM function led to substantial deregulation of *Brca1* expression, while *Rb*^*-/-*^ cells showed a weaker effect. Complete deregulation, with similar expression levels in G_0_ and S phase, was observed for both *Brca1* and *Brca2* in DKO cells. Additionally, overall expression levels in knockout cells were consistently higher than in wild-type cells, indicating a general loss of repression in these mutants. Rescue experiments confirmed the significant role of Lin37/DREAM in repressing *Brca2* and, particularly, *Brca1* in G_0_ and the early phases of the cell cycle (Fig. S2).

In summary, these results indicate that the DREAM and Rb complexes are essential for the repression of *Brca1* and *Brca2* in G_0_-arrested cells and during the early cell cycle. The DREAM complex appears to contribute more substantially to the repression of *Brca1* compared to Rb:E2f complexes.

### Activation of *BRCA1* and *BRCA2* transcription is not mediated by MuvB-based complexes

Given that DREAM is involved in the downregulation of *BRCA1* and *BRCA2* expression, we also investigated whether their transcriptional activation is mediated by the corresponding MuvB-based complexes [29]. Knockdown of A-MYB and B-MYB did not significantly affect *BRCA1* and *BRCA2* mRNA expression (Fig. S3). These results suggest that transcriptional activation of *BRCA1* and *BRCA2* by A-MYB:MuvB or B-MYB:MuvB complexes does not contribute to the regulation of these genes, despite the clear role of the DREAM repressor in their downregulation. Furthermore, these results indicate that CHR promoter elements are unlikely to be involved in *BRCA1/2* gene regulation, consistent with the characterization of *BRCA1/2* as early cell cycle genes exhibiting maximal expression during S phase [28].

### E2F sites regulate cell cycle-dependent *BRCA1* and *BRCA2* transcription

**Fig. 3:**
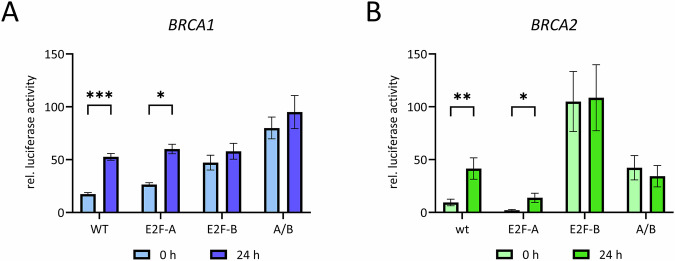
Cell cycle-dependent transcription of *BRCA1* and *BRCA2* depends mostly on their proximal E2F-B sites. NIH3T3 cells were transfected with luciferase reporter constructs of the wild-type human promoters (wt) and mutant promoters for potential transcription factor binding sites (E2F-A, E2F-B, and A/B) of (**A**) *BRCA1*, with A/B representing a mutant of E2F-A and E2F-B or (**B**) wt and mutant promoter construct of *BRCA2* together with a *Renilla* luciferase control reporter plasmid. Cells were synchronized in G_0_ by serum starvation, stimulated to re-enter the cell cycle be serum addition and collected after 24 h. Relative luciferase activity is given (Mean ± SEM, n = 3–4; two-tailed unpaired t test; *p ≤ 0.05, **p ≤ 0.01, ***p ≤ 0.001).

To investigate the role of the conserved putative E2F binding sites (Fig. 1D) in regulating *BRCA1* and *BRCA2* transcription, we amplified the regions surrounding the transcription start sites to construct luciferase reporter plasmids for the two human genes. Repression of the *BRCA1* gene in G_0_ was primarily mediated by the proximal E2F-B site, whereas inactivation of the E2F-A site resulted in only minor derepression of the promoter (Fig. 3A). The E2F-B site is in reverse orientation and is located proximally upstream of the transcriptional start site (Fig. 1D).

Similarly, we analyzed the putative E2F binding sites in the *BRCA2* promoter using luciferase reporter assays. The proximal E2F-B site in the *BRCA2* promoter was identified as critical for G_0_-specific repression, while the distal E2F-A element had no substantial regulatory role in the cell cycle (Fig. 3B).

### DREAM and RB:E2F complexes bind to the *BRCA1* and *BRCA2* promoters differentially in the cell cycle

**Fig. 4:**
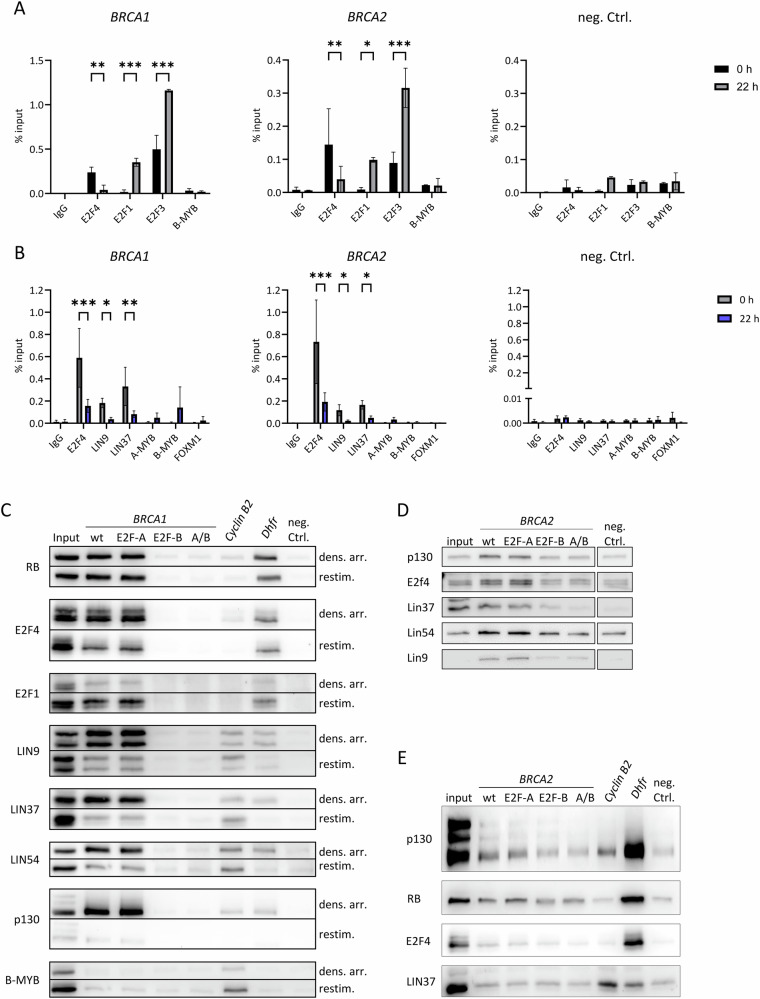
DREAM and RB:E2F complexes bind to the *BRCA1* and *BRCA2* promoters. **A** Chromatin immunoprecipitations (ChIPs) were performed with cross-linked chromatin from serum-starved (0 h) or restimulated (22 h) T98G cells. Antibodies targeted E2F4, E2F1, E2F3, or B-MYB. A non-targeting antibody (IgG) and the promoter of the *GAPDHS* gene served as a negative control. The *BRCA1* and *BRCA2* promoters were detected by real-time qPCR. All signals are given relative to the input DNA signal. **B** ChIPs were performed with cross-linked chromatin from starved (0 h) or restimulated (22 h) RPE-1 cells. Antibodies targeted E2F4, LIN9, LIN37, A-MYB, or B-MYB. A non-targeting antibody (IgG) and the promoter of the *GAPDHS* gene served as a negative control. The *BRCA1* and *BRCA2* promoters were detected by real-time qPCR. All signals are given relative to the input DNA signal. **A**, **B** Mean ± SD; two-way ANOVA; n = 3; *p ≤ 0.05; **p ≤ 0.01; ***p ≤ 0.001. **C** Nuclear extracts of density-arrested RPE-1 cells and cells restimulated for 20 h were employed for DNA affinity purification using biotinylated wt and mutant (E2F-A, E2F-B, and A/B) *BRCA1* promoter probes and a promoter probe of the late cell cycle gene *Cyclin B2* as well as a mouse promoter probe of the early cell cycle gene *Dhfr*. As a negative control, a fragment of the mouse *Gapdhs* promoter (neg. Ctrl.) was used. **D** DREAM components (p130, E2f4, Lin37, Lin54, and Lin9) were purified from nuclear extracts of density-arrested NIH3T3 mouse cells by DNA affinity purification and detected by Western blot. Binding to the human *BRCA2* wild-type promoter probe (wt) was compared with binding to mutant probes (E2F-A, E2F-B, and A/B). Background protein binding was determined with a probe of the mouse *cyclin B2* CHR mutant promoter (neg. Ctrl.). **E** p130, RB, E2F4, and LIN37 were purified from nuclear extracts of proliferating HCT116 human cells by DNA affinity purification and detected by Western blot. Binding to the wild-type promoter probe (wt) was compared with binding to E2F site mutant probes (E2F-A, E2F-B, and A/B) as well as binding to the promoter of the late cell cycle gene *Cyclin B2* and the mouse promoter of the early cell cycle gene *Dhfr*. As a negative control, binding to a fragment of the mouse *Gapdhs* promoter was tested (neg. Ctrl.).

We analyzed the binding of DREAM and RB:E2F complexes in living cells using chromatin immunoprecipitation (ChIP) assays and in vitro through DNA affinity purification. Additionally, we distinguished between binding events in resting cells (G_0_) and in restimulated proliferating cells. We found that the binding patterns are similar between the *BRCA1* and *BRCA2* genes (Fig. 4).

ChIP assays demonstrated that the DREAM repressor subunits E2F4, LIN9, and LIN37 preferentially bind to *BRCA1* and *BRCA2* promoter regions in G_0_-arrested cells (Fig. 4A, B). In contrast, the transcription factors E2F1 and E2F3, which are associated with gene activation, predominantly bind to these promoters in restimulated cells. The oncogenic transcription factors A-MYB, B-MYB, and FOXM1, which require MuvB complex formation to bind through CHR promoter elements, do not show significant binding to either *BRCA1* or *BRCA2* promoter regions (Fig. 4A, B).

In vitro binding assays are consistent with the ChIP data obtained from G_0_ and restimulated cells (Fig. 4). Components of both RB:E2F complexes (RB and E2F1) and DREAM complexes (E2F4, LIN9, LIN37, LIN54, and p130) bind specifically through the E2F-B site in the *BRCA1* promoter. In contrast, the E2F-A site is not required for binding. Furthermore, the CHR-dependent activator B-MYB does not exhibit significant binding to the *BRCA1* probe in these assays (Fig. 4C). Assessing protein binding to the *BRCA2* probe in this in vitro assay proved challenging. Despite this limitation, subtle differences in binding among the various probes could be observed, indicating that for *BRCA2*, the E2F-B site - and not the E2F-A site - is the primary binding site for RB:E2F and DREAM complexes (Fig. 4D, E). Taken together, the in vitro binding data are consistent with the ChIP data (Fig. 4A, B).

Furthermore, ChIP-seq data corroborate the binding of RB and DREAM components to the *BRCA1* and *BRCA2* promoter loci (Fig. S4).

### p53 downregulates *BRCA1* and *BRCA2* expression indirectly via inducing expression of p21/CDKN1A

**Fig. 5:**
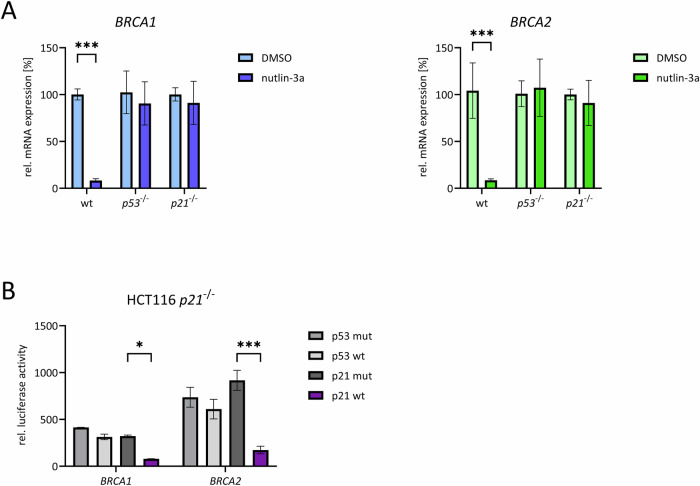
p53 downregulates *BRCA1* and *BRCA2* expression indirectly via inducing expression of p21/CDKN1A. **A** p53-positive wild-type (*p53*^*+/+*^) or p21-negative (*p21*^*-/-*^) HCT116 cells were treated for 48 h with nutlin-3a, or DMSO as control. Expression levels of mRNAs were determined by qPCR (Mean ± SD, n = 3, two-way ANOVA; ***p ≤ 0.001). **B** HCT116 *p21*^*-/-*^ cells were transfected with wild-type (wt) *BRCA1* or *BRCA2* promoter reporter constructs together with expression plasmids for wild-type or mutant variants of p53 (p53 wt; p53 mut) or p21 (p21 wt; p21 mut). 24 h after transfection, promoter reporter activities were analyzed by luciferase assays with relative luciferase activity calculated as the ratio of firefly luciferase activity from the promoter reporter constructs to *Renilla* luciferase activity from a cotransfected control plasmid lacking a promoter (Mean ± SD, n = 2 to 4; two-way ANOVA; *p ≤ 0.05; ***p ≤ 0.001).

To investigate the response of *BRCA1* and *BRCA2* expression to apoptosis induction and cell cycle arrest, we treated cells with DNA-damaging agents or induced p53 stabilization (Fig. 5). We observed that *BRCA1* and *BRCA2* mRNA expression was downregulated upon treatment with nutlin-3a. Notably, this downregulation was abolished in cells with deletions of the CDK inhibitor p21/CDKN1A (Fig. 5A).

To further assess whether the p53-dependent downregulation relies on transcriptional regulation and can be rescued by p21 re-expression in p21-deficient cells, we conducted experiments using *BRCA1* and *BRCA2* promoter reporter constructs. Cotransfection of expression plasmids for wild-type or mutant variants of p53 or p21 into HCT116 *p21*^*-/-*^ cells demonstrated that p53 alone, without subsequent p21 expression, is insufficient to induce *BRCA1* and *BRCA2* downregulation. However, re-expression of p21 in a p21-deficient background resulted in the downregulation of *BRCA1* and *BRCA2* expression, even in the absence of p53 induction (Fig. 5B).

### LIN37/DREAM and RB cooperate in p53/p21-dependent *BRCA1* and *BRCA2* repression

**Fig. 6:**
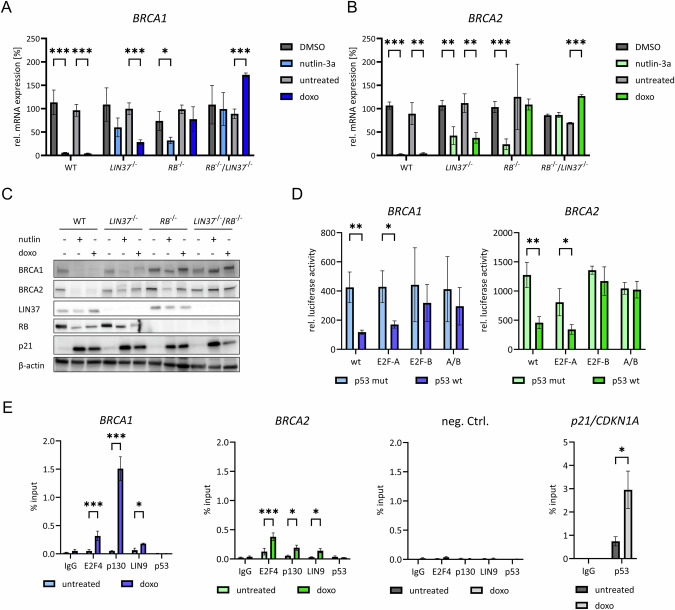
LIN37/DREAM and RB mediate p53/p21-dependent downregulation of *BRCA1* and *BRCA2.* **A**,** B** Gene expression of *BRCA1* or *BRCA2* in HCT116 wild-type (WT) and mutant cells were tested. Clonal cell lines for WT n = 4, *LIN37*^*−/−*^ n = 4, *RB*^*−/−*^ n = 3, or DKO *LIN37*^*−/−*^*; RB*^*−/−*^ n = 2 were treated with nutlin-3a or doxorubicin for 48 h. Controls were untreated or DMSO-treated (solvent control) for 48 h. *BRCA1* or *BRCA2 mRNA levels* were determined by real-time RT-qPCR. Mean values ± SD from two technical replicates in the allocated number of clones are shown. Significances were calculated using the Student’s t-test (n.s., not significant; *p  ≤  0.05; **p  ≤  0.01; ***p  ≤  0.001). **C** Immunoblot detection of BRCA1 or BRCA2 protein from protein extracts of one representative clone of each genotype from the same experiment described in (**A**, **B**). For knockout confirmation LIN37 and RB protein levels are shown. p21 protein levels were analyzed for p53 activation. β-Actin served as loading control. 10 µg whole RIPA protein extract were used. **D** HCT116 *p53*^*-/-*^cells were transfected with wild-type (wt) and mutant (E2F-A, E2F-B, and A/B) *BRCA1* or *BRCA2* promoter reporter constructs together with a *Renilla* luciferase control plasmid and with expression plasmids for p53 wild-type (p53 wt) or a DNA binding-deficient mutant (p53 mut). 24 h after transfection, promoter reporter activities were analyzed by luciferase assays and are given as relative luciferase activity. (Mean ± SEM, n = 3 to 5; two-way ANOVA; *p ≤  0.05, **p  ≤  0.01). **E** Chromatin immunoprecipitations were performed with cross-linked chromatin from untreated or doxorubicin-treated HCT116 wild-type cells. Antibodies targeted p130, E2F4, LIN9, or p53. The *p21/CDKN1A* gene served as a positive control for p53 binding. A non-targeting antibody (IgG) served as a negative control. The indicated promoters were detected by real-time qPCR. All signals are given relative to the input DNA signal (Mean ± SEM, n = 2 to 5; two-way ANOVA; *p ≤ 0.05, ***p ≤ 0.001).

Next, we investigated the respective contributions of LIN37/DREAM and RB to the p53/p21-dependent downregulation of BRCA1 and BRCA2. Upon p53/p21 induction in wild-type HCT116 cells following treatment with nutlin-3a or doxorubicin, *BRCA1* and *BRCA2* mRNA expression was strongly downregulated (Fig. 6A, B). This repression was largely abolished in cells deficient for either LIN37/DREAM or RB. Notably, in double-knockout cells lacking both LIN37 and RB, the downregulation was completely abrogated. Furthermore, BRCA1 and BRCA2 protein expression mirrored the corresponding mRNA levels, confirming that transcriptional repression directly translates to reduced protein abundance (Fig. 6C).

### p53-dependent transcriptional repression requires the proximal E2F sites in the *BRCA1* and *BRCA2* promoters and DREAM binding

We next investigated which promoter elements mediate the indirect transcriptional repression following p53 induction. To this end, wild-type and mutant *BRCA1* and *BRCA2* promoter constructs with inactivated E2F sites were analyzed in p53-deficient HCT116 cells. Consistent with the results presented in Fig. 3, downregulation of the promoters upon overexpression of wild-type p53 primarily depends on the proximal E2F-B sites in both genes (Fig. 6D).

Using ChIP assays, we demonstrated that the DREAM complex ─ represented by the subunits E2F4, p130, and LIN9 ─ binds to the *BRCA1* and *BRCA2* promoter regions following p53 induction by doxorubicin treatment in HCT116 cells (Fig. 6E). Notably, LIN9 binding to both promoters was significantly reduced in cells transfected with an inactive p53 mutant compared to those expressing wild-type p53. We tested LIN9 binding as a representative subunit of the MuvB core complex, which is a component of both the DREAM repressor complex and the MuvB activator complexes (A-MYB:MuvB, B-MYB:MuvB, and FOXM1:MuvB) [29, 31, 34]. Thus, when LIN9 binding is absent on promoters that are active and capable of DREAM binding when repressed, these promoters contain functional E2F sites but lack CHR elements. These findings support our earlier observation (Fig. S3) that MuvB activator complexes, which require CHR sites for promoter binding, do not activate *BRCA1* and *BRCA2* expression.

Therefore, these results support our earlier finding (Fig. S3) that MuvB activator complexes, which require CHR sites for binding, do not activate *BRCA1* and *BRCA2* expression.

Furthermore, we did not observe significant p53 binding to the *BRCA1* and *BRCA2* genes, whereas p53 binding to the *p21/CDKN1A* promoter was markedly increased (Fig. 6E). These observations show that p53-dependent downregulation of *BRCA1* and *BRCA2* is not caused by direct p53 binding to the promoters but is indirect.

## Discussion

BRCA1 and BRCA2 exert central functions in DNA repair by homologous recombination (HR). The proteins form a complex and cooperate functionally [3, 14–18]. Their cooperation requires a coordinated expression of the two proteins. Remarkably, we find that a coordinated expression is achieved by controlling *BRCA1* and *BRCA2* gene expression by essentially identical transcriptional mechanisms. These common mechanisms regulate parallel expression during the cell cycle and indirect downregulation by p53.

To investigate the role of DREAM and RB in the cell cycle-dependent expression of *BRCA1* and *BRCA2*, we utilized our Lin37/DREAM and Rb knockout mouse cell models. Our findings indicate that the deletion of *Lin37* or *Rb* leads to the deregulation of both *Brca1* and *Brca2* mRNA expression. Notably, *Brca1* deregulation is more pronounced in *Lin37*^−/−^ cells compared to *Rb*^−/−^ cells. Complete deregulation of both genes is observed in *Lin37*^−/−^*;Rb*^−/−^ double-knockout cells.

Since the DREAM repressor can switch into the A/B-MYB:MuvB and FOXM1:MuvB activator complexes, we also investigated whether *BRCA1/2* expression is regulated by MuvB-based complexes through A-MYB or B-MYB knockdown. Our results demonstrate that neither *BRCA1* nor *BRCA2* is activated by A-MYB or B-MYB. This finding aligns with the absence of CHR promoter sites in the *BRCA1* and *BRCA2* promoters, which are necessary for this mode of activation [26, 28–30, 32, 34, 51].

Regarding the regulatory elements involved in cell cycle-dependent transcription, we identified two conserved E2F sites, with the respective proximal elements playing the most significant role in promoter reporter assays for both *BRCA1* and *BRCA2* genes.

To further examine protein binding to these genes, we performed ChIP and DNA affinity purification assays. Our findings indicate that DREAM components, including E2F4, preferentially bind to the *BRCA1* and *BRCA2* promoters in quiescent cells. Consistent with our functional data, this binding predominantly occurs at the proximal E2F sites within their respective promoters. Similarly, RB binding to the proximal E2F sites in both promoters is preferentially observed in quiescent cells, as demonstrated by DNA affinity purification. In restimulated cells, the RB binding partner E2F1 is recruited to the promoters, whereas E2F3 exhibits binding in both quiescent and restimulated cells, albeit with increased association upon restimulation.

The formation of DREAM and RB:E2F repressor complexes results from the inhibition of cyclin/CDK activity, which induces a switch from hyperphosphorylated to hypophosphorylated RB and the RB-related proteins p107 (*RBL1*) and p130 (*RBL2*), subsequently leading to repressor complex assembly [25, 34]. Regarding the upregulation of *BRCA1* by cyclin/CDK activity, previous studies have shown that overexpression of Cyclin D1 and CDK4 activates the mouse *Brca1* promoter via the conserved E2F-B promoter site [40]. These findings align with our conclusions but were originally interpreted — prior to the discovery of DREAM — as being dependent on RB:E2F complexes. In contrast, our results demonstrate that *BRCA1* repression is governed by DREAM and RB.

**Fig. 7:**
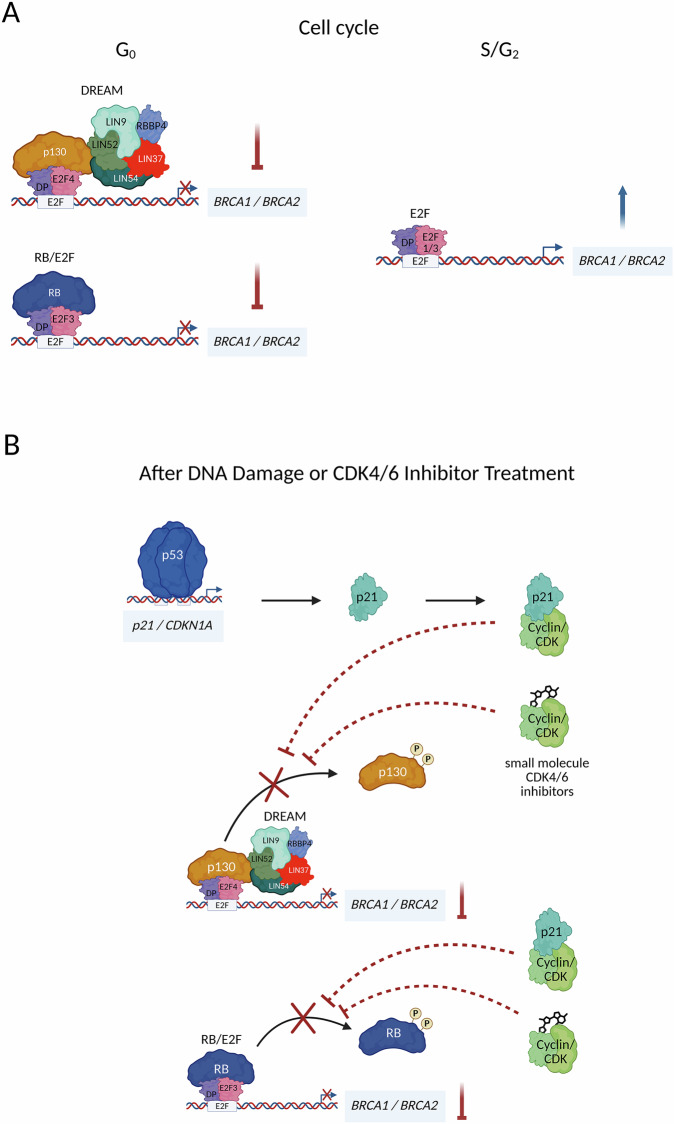
Regulation of *BRCA1* and *BRCA2* expression during the cell cycle and in response to DNA damage or inhibitor treatment. Expression of *BRCA1* and *BRCA2* is primarily regulated through a shared mechanism. **A** During G_0_, transcription is repressed by DREAM and RB:E2F repressor complexes binding to E2F promoter sites. In S/G_2_ phases, the loss of these repressor complexes allows activating E2Fs to bind the promoters, thereby promoting gene expression. **B** Following DNA damage, the same complexes mediate downregulation of *BRCA1* and *BRCA2*. DREAM and RB:E2F repressor complexes are formed when cyclin/CDK complexes are inhibited by p21, whose expression is induced by p53 activation. Thus, p53 indirectly represses *BRCA1* and *BRCA2* transcription via p21-mediated CDK inhibition. In a therapeutic context, small molecule CDK4/6 inhibitors such as ribociclib, palbociclib, and abemaciclib can functionally mimic p21 to suppress *BRCA1* and *BRCA2* expression. The figure was created using BioRender.com.

These findings highlight the role of DREAM in BRCA1 regulation and suggest a broader mechanism governing the coordinated expression of *BRCA1* and *BRCA2* during the cell cycle. Based on our results, we propose a model in which transcriptional complexes bind to the *BRCA1* and *BRCA2* promoters in a nearly identical manner, explaining their synchronized expression with highly similar timing throughout the cell cycle (Fig. 7A). In quiescent cells, DREAM and RB:E2F complexes compete for binding at the same E2F site to repress the *BRCA1* and *BRCA2* promoters. In proliferating cells, the DREAM complex dissociates from the E2F site, allowing E2F1/3:DP complexes to bind to the proximal E2F sites in both promoters and activate transcription.

BRCA1 and BRCA2 functionally and physically interact to regulate key pathways in the DNA damage response (DDR) and cell cycle checkpoint control [3, 12, 14, 17]. These proteins link DNA damage sensing with repair initiation, primarily facilitating HR in DSB repair [22]. DNA repair pathway choice depends on the cell cycle phase: HR predominates in S and G_2_ phases, while NHEJ is active in G_1_ phase. In the absence of functional BRCA1 and BRCA2, error-prone pathways like NHEJ and SSA are utilized, increasing chromosomal aberrations [22]. The crucial role of BRCA1 and BRCA2 in genome stability is evident in conditional *Brca1*-mutant mice, which exhibit severe genomic instability and aneuploidy [52]. Additionally, BRCA1 and BRCA2 stabilize stalled replication forks during S phase and contribute to spindle assembly checkpoint (SAC) regulation and telomere maintenance in later cell cycle stages [3, 14, 15, 53].

Consistent with these roles, BRCA1 and BRCA2 are predominantly expressed in S phase, forming multi-protein complexes essential for HR. While BRCA1 possesses E3 ubiquitin ligase activity in complex with BARD1 [3], both proteins rely on key interaction partners, including RAD51, PALB2, CHK2, CDK2, and PLK1, which regulate DNA repair and cell cycle progression [35, 54]. Notably, BRCA1 (*FANCS*) and BRCA2 (*FANCD1*) belong to the Fanconi anemia complementation group, which preserves genomic integrity by repairing DNA interstrand crosslinks and stabilizing replication forks [22, 55]. Several BRCA1/2 interactors, such as PALB2 (*FANCN*), RAD51 (*FANCR*), and BRIP1 (*FANCJ*), are also Fanconi anemia proteins [56, 57].

Importantly, *BRCA1, BRCA2*, and several associated genes — particularly members of the Fanconi anemia gene family — are transcriptionally regulated by the DREAM complex [20]. DREAM controls multiple DNA repair genes, including *PALB2, RAD51*, and *BRIP1*, as well as cell cycle regulators *CDK1 (CDC2), Cyclin A*, and *PLK1* — essential for BRCA2-mediated cytokinesis [20, 21, 28, 34, 49, 54]. In quiescent cells, DREAM-mediated repression of HR genes prevents untimely repair [58, 59]. Additionally, BRCA1-BARD1 complexes regulate the G_2_/M checkpoint by ubiquitinating Cyclin B and CDC25C — both DREAM/MuvB-regulated genes — facilitating controlled cell cycle progression [26, 49, 60].

Further examples of DREAM-dependent regulation include Ki-67, a mitotic chromosome surfactant, and PLK4, a centriole biogenesis regulator, both co-functioning with BRCA1/2 in mitosis and cytokinesis [48, 61, 62]. Importantly, these findings highlight the co-regulation and the extensive interplay between BRCA1/2 and DREAM in coordinating DNA repair, cell cycle checkpoints, and genomic stability.

Taken together, DREAM and RB:E2F complexes orchestrate the transcriptional repression of *BRCA1*, *BRCA2*, and their co-factors in G_0_ and early G_1_ phases, followed by strong induction in S and G_2_ phases. This precise temporal regulation ensures their coordinated expression, reinforcing their critical roles in DNA repair, cell cycle progression, and genomic stability.

In addition to investigating transcriptional regulation during the cell cycle, we examined the control of *BRCA1* and *BRCA2* expression following DNA damage induction. Upon DNA damage, p53 downregulates *BRCA1* and *BRCA2* expression without directly binding to their promoters. We observed that, in addition to p53, the CDK inhibitor p21 is also required for *BRCA1*/*BRCA2* repression.

Notably, DNA damage and p53 activation also drive cells out of S phase — the cell cycle phase in which BRCA1 and BRCA2 exert their functions [37]. Consequently, two regulatory mechanisms work in parallel to restrict BRCA1/BRCA2 function: transcriptional downregulation and cell cycle arrest at either the G_1_/S or G_2_/M checkpoints.

Regarding the requirement for transcription factors, co-factors, and their binding sites in the promoters, our findings on p53-induced repression align with those from cell cycle-dependent regulation. Both LIN37/DREAM and RB are essential for DNA damage-induced repression, which is predominantly mediated through the proximal E2F sites in the *BRCA1* and *BRCA2* promoters. The binding of the representative DREAM components E2F4, p130, and LIN9 to both promoters is enhanced upon DNA damage.

Previous studies had observed that p53 downregulates *BRCA1* protein and mRNA expression before initiating p53-dependent cell cycle arrest and apoptosis, though the underlying mechanism was not elucidated [63]. Similarly, *BRCA2* mRNA levels and promoter activity have been shown to be repressed following p53 induction. However, one study attributed *BRCA2* regulation to transcriptional activation by USF1 or USF2, which contradicts the findings presented here [64]. Additionally, a recent report proposed an alternative, non-canonical mechanism of p53- and DREAM-dependent gene repression, in which p53 binds directly to target promoters without requiring p21 as a mediator [65]. In contrast, our study observed no significant binding of p53 to the *BRCA1* and *BRCA2* promoters. Moreover, the downregulation of these genes requires both p53 and p21, thus supporting their regulation via the canonical p53-p21-DREAM pathway [34].

Taken together, our results support a mechanism by which p53 indirectly downregulates *BRCA1* and *BRCA2* transcription (Fig. 7B). Activated p53 directly transactivates *p21/CDKN1A*, leading to increased expression of the p21 CDK inhibitor. Elevated p21 levels result in the hypophosphorylation of p107, p130, and RB promoting the formation of DREAM and RB:E2F complexes. These complexes subsequently bind to E2F sites in the *BRCA1* and *BRCA2* promoters, leading to gene repression. Thus, the p53-p21-DREAM/RB pathways provide a mechanistic link between p53 activation and the indirect downregulation of *BRCA1* and *BRCA2* expression (Fig. 7B). This regulatory mechanism is consistent with the transcriptional control of many other genes, including those encoding *BRCA1*/*BRCA2* interactors, which are governed by the p53-p21-DREAM/RB pathways [25, 34].

An important clinical implication of *BRCA1*/*2* regulation by the p53-p21-DREAM/RB pathways arises from the therapeutic potential of cyclin-dependent kinase (CDK) inhibitors. The CDK4/6 inhibitors palbociclib, abemaciclib, ribociclib, and trilaciclib functionally overlap with the inhibitory role of p21/CDKN1A [66, 67]. Consequently, in tumors lacking functional p53, these inhibitors can compensate for the loss of p21 induction, leading to the downregulation of BRCA1 and BRCA2 expression by blocking CDK4/6 activity and promoting DREAM and RB:E2F complex formation [25, 34] (Fig. 7B).

In general, there are two key functional properties of BRCA1 and BRCA2 that are not fully understood. First, their role as tumor suppressors — primarily in hereditary breast and ovarian cancer — remains elusive. It is unclear why these proteins fail to exert tumor-suppressive functions in breast and ovarian cancers driven by somatic mutations, or why they do not effectively suppress tumor formation in other tissues, despite their ubiquitous expression and fundamental role in DNA repair. A recent review highlights that this cancer tissue tropism remains an unresolved enigma [68]. One possible explanation is that hormonal responsiveness of breast and ovarian tissues contributes to their susceptibility. Moreover, the limited tumor-suppressive activity of BRCA1 and BRCA2 in sporadic breast and ovarian cancers may result from the relatively late occurrence of somatic mutations, in contrast to inherited mutations, which are present throughout development and provide more time for oncogenic processes to accumulate.

The second enigma is the seemingly paradoxical interaction between tumor suppressors, specifically the indirect repression of *BRCA1* and *BRCA2* by p53. It is unclear how this downregulation contributes to tumor suppression, as one would not typically expect a tumor suppressor to inhibit the function of another tumor suppressor. A key to addressing this question lies in understanding the roles of BRCA1 and BRCA2 in DNA synthesis and repair, and ultimately, their indirect contribution to the induction of cell death. BRCA1 and BRCA2 play crucial roles in the repair of DSBs through HR [3]. Loss or downregulation of BRCA1 or BRCA2 impairs the cell’s ability to perform error-free DNA repair via HR, thereby forcing reliance on error-prone pathways such as non-homologous end joining (NHEJ) and single-strand annealing (SSA). The resulting repair errors are thought to contribute to malignant transformation. However, counterintuitively, defects in DNA repair — such as those arising from impaired HR due to BRCA1 or BRCA2 loss — can trigger cell cycle arrest and ultimately induce cell death [9, 22, 69]. Therefore, reduced BRCA1 and BRCA2 expression regulated by p53 may, in fact, represent a tumor-suppressive mechanism. The concept — that the loss of BRCA1 or BRCA2 can act as a trigger for cell death and thereby contribute to tumor suppression — has been proposed previously [9, 69].

A significant consequence of the coordinated, cell cycle-dependent expression of BRCA1 and BRCA2, alongside their interaction partners, is that DSB repair by HR is tightly restricted to S and G_2_ phases, whereas DNA repair through NHEJ predominates in quiescent and G_1_ cells [3, 9, 14–18, 22, 70]. This regulation ensures that HR occurs only when a homologous sister chromatid is available, minimizing the risks associated with imprecise repair mechanisms. The importance of this regulatory control is underscored by experiments showing that DREAM, by downregulating BRCA1, BRCA2, and other factors, helps prevent excessive DNA end resection and aberrant HR-mediated DSB repair in G_0_ cells [59].

Consequently, the lack of BRCA1 and BRCA2 expression, whether due to cell cycle regulation or p53-mediated downregulation, causes a shift from HR to error-prone NHEJ, thereby promoting genomic instability. The resulting accumulation of genomic defects can ultimately trigger cell death. This regulatory mechanism may provide insight into why p53, as a tumor suppressor, downregulates factors that are also considered tumor suppressors – albeit only in specific settings as in hereditary ovarian and breast cancer.

Collectively, our findings provide mechanistic insight into the stress- and cell cycle-dependent downregulation of BRCA1 and BRCA2, highlighting how their reduced expression, likely occurring across all tissues, shifts DNA repair toward error-prone pathways and promotes cell death as a tumor-suppressive response.

In summary, our results demonstrate that p53/p21-dependent transcriptional repression of *BRCA1* and *BRCA2* requires DREAM and RB. This regulation — as well as cell cycle-dependent control — relies on DREAM and RB:E2F binding to proximal E2F sites in the promoters of these genes. Therefore, DREAM- and RB-mediated transcriptional control of *BRCA1* and *BRCA2* constitutes a critical component of the regulatory network governing DNA repair, cell survival, and tumor suppression.

## Supplementary information

Suppl. Fig. S1

Legend Suppl. Fig. S1

Suppl. Fig. S2

Legend Suppl. Fig. S2

Suppl. Fig. S3

Legend Suppl. Fig. S3

Suppl. Fig. S4

Legend Suppl. Fig. S4

Suppl. Fig. Uncropped Westerns

## Data Availability

All data supporting the findings of this study are available within the paper and its Supplementary Information.
