# Supplementary material for: BRCA1 and BRCA2 gene expression: p53- and cell cycle-dependent repression requires RB and DREAM
Source: Cell Death Differ. 2025 Aug 22;33(1):51–63. doi: 10.1038/s41418-025-01566-9 (PMC12811384; doi:10.1038/s41418-025-01566-9)
Supplement: Supplementary file 2 — Legend Suppl. Fig. S1 [file 41418_2025_1566_MOESM2_ESM.docx]

**Legend Suppl. Fig. S1**

***BRCA1* and *BRCA2* mRNA is maximally expressed during S phase of the cell cycle. (A)** *BRCA1* and *BRCA2* mRNA expression normalized to U6 RNA expression was measured in serum-starved and restimulated HFF cells and compared to the early cell cycle gene *DHFR* and the late cell cycle gene *BUB1* (n=4). **(B)** As a control for cell cycle distribution in (A), DNA content staining was analyzed by flow cytometry and cells were grouped into G_0_/G_1_, S or G_2_/M phase based on their DNA content. **(C)** *Brca1* and *Brca2* mRNA expression normalized to U6 RNA expression was measured in density-arrested and released NIH3T3 cells and compared to a set of early cell cycle genes (*Cdc6* and *Dhfr*) as well as a set of late cell cycle genes (*Bub1* and *Ccnb2*, n≥3). **(D)** As a control for cell cycle distribution in (C), DNA content staining was analyzed by flow cytometry and cells were grouped into G_0_/G_1_, S or G_2_/M phase based on their DNA content. Mean ± SD and sixth order polynomial regressions with 95% CI are given.
