# Supplementary figures and images for: BRCA1 and BRCA2 gene expression: p53- and cell cycle-dependent repression requires RB and DREAM
Source: Cell Death Differ. 2025 Aug 22;33(1):51–63. doi: 10.1038/s41418-025-01566-9 (PMC12811384; doi:10.1038/s41418-025-01566-9)

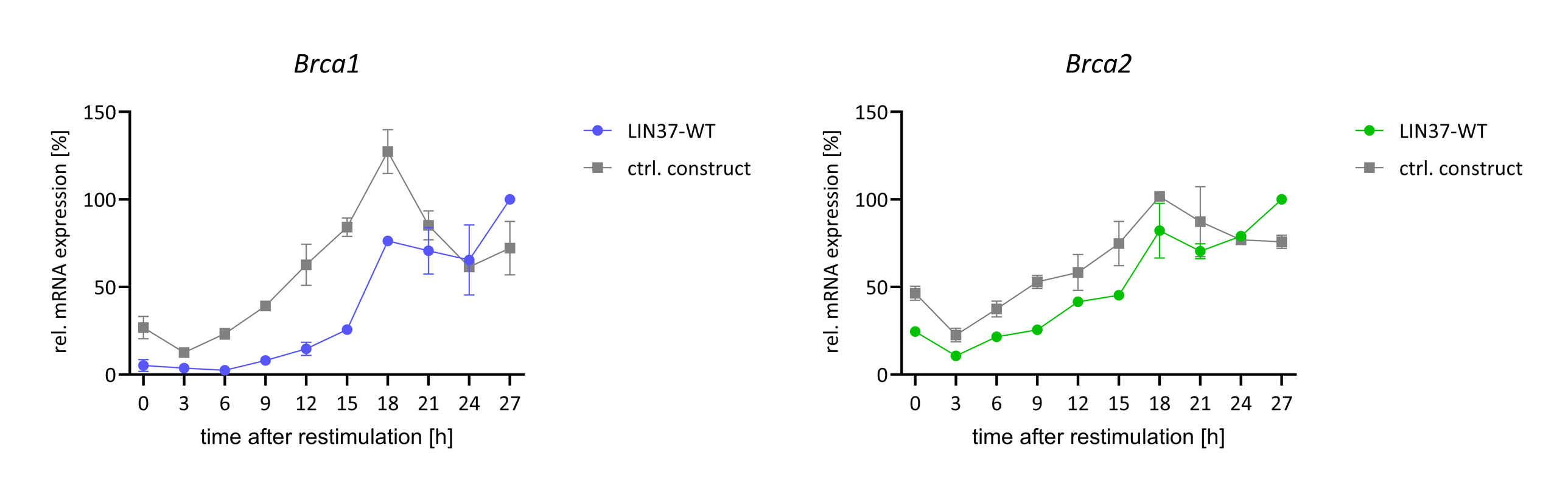

Supplement: Supplementary file 3 — Suppl. Fig. S2 [file 41418_2025_1566_MOESM3_ESM.tif]

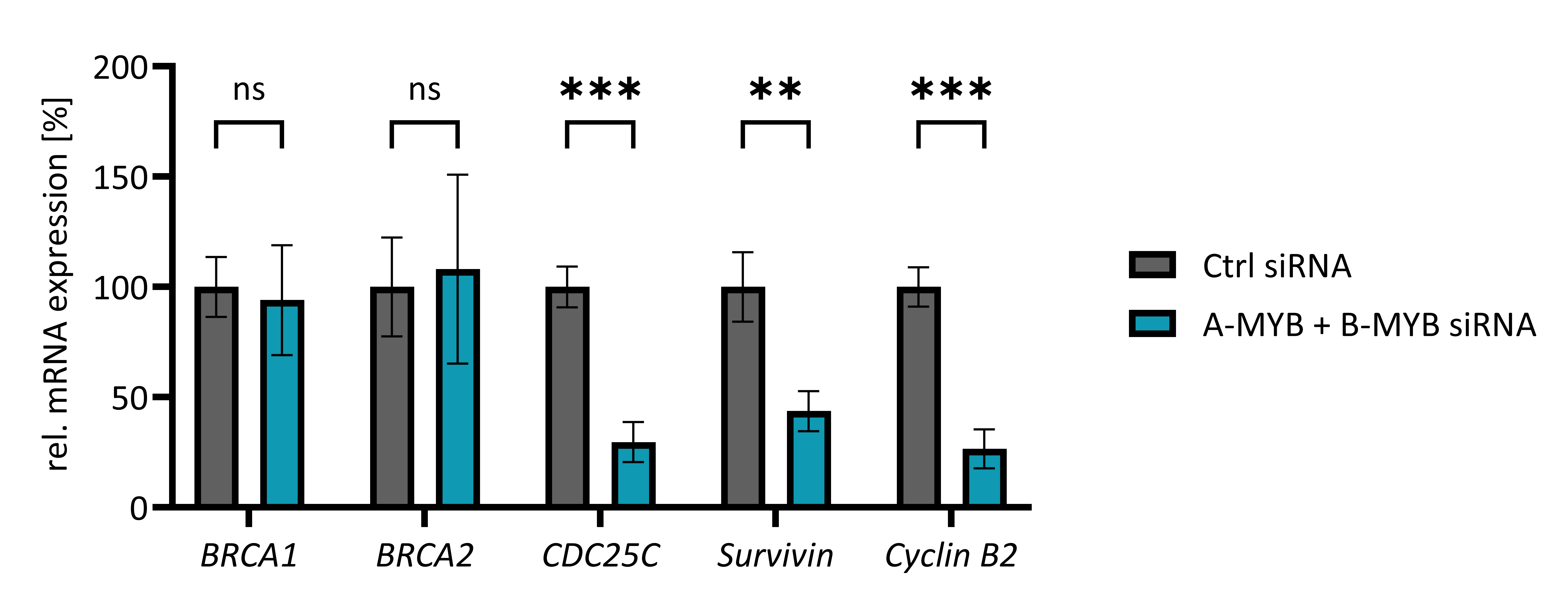

Supplement: Supplementary file 5 — Suppl. Fig. S3 [file 41418_2025_1566_MOESM5_ESM.tif]

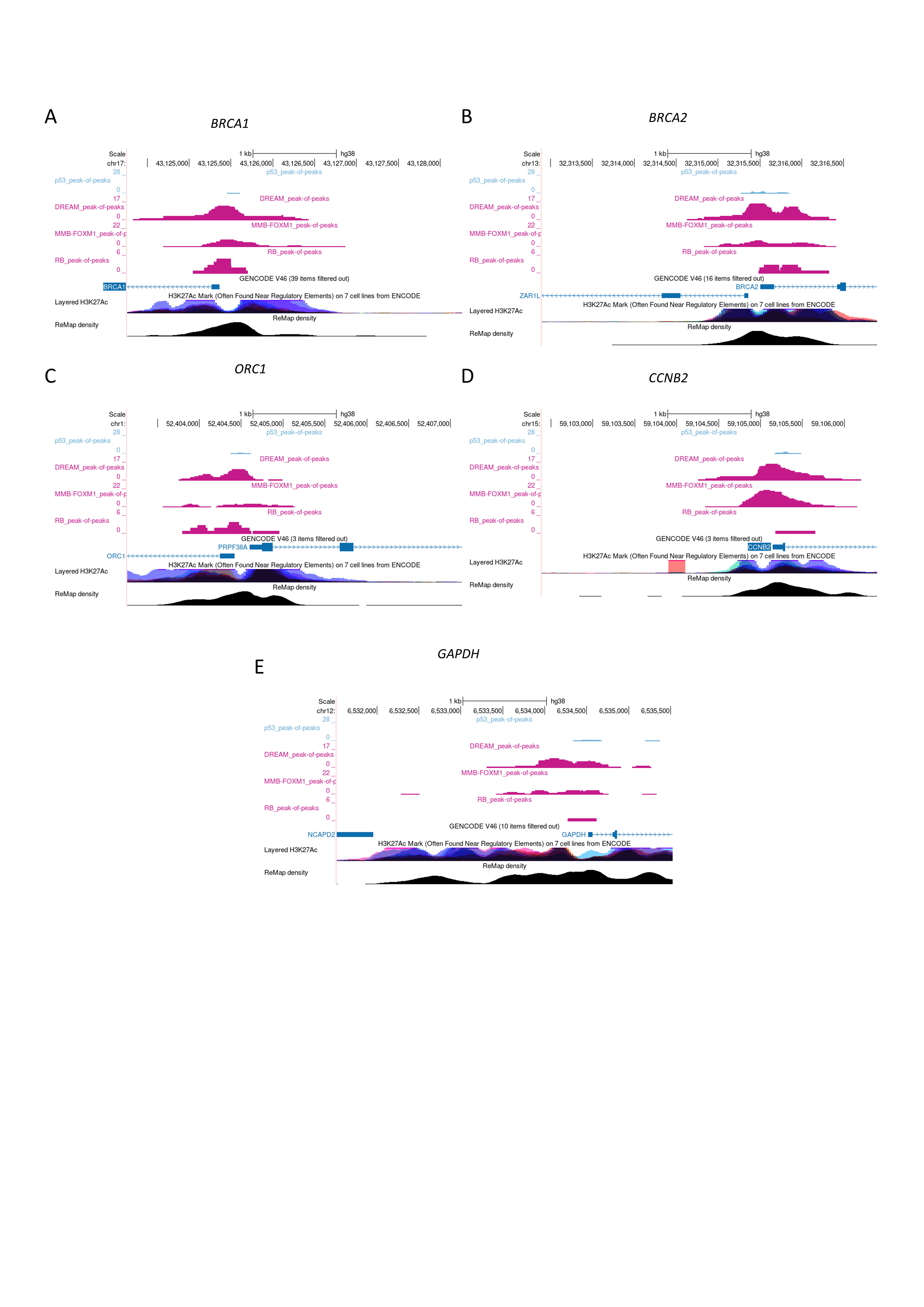

Supplement: Supplementary file 7 — Suppl. Fig. S4 [file 41418_2025_1566_MOESM7_ESM.tif]
