# Supplementary material for: BRCA1 and BRCA2 gene expression: p53- and cell cycle-dependent repression requires RB and DREAM
Source: Cell Death Differ. 2025 Aug 22;33(1):51–63. doi: 10.1038/s41418-025-01566-9 (PMC12811384; doi:10.1038/s41418-025-01566-9)
Supplement: Supplementary file 4 — Legend Suppl. Fig. S2 [file 41418_2025_1566_MOESM4_ESM.docx]

**Legend Suppl. Fig. S2**

**Rescue of *Brca1* and *Brca2* downregulation with expression of wild-type Lin37 in *Lin37-/-* cells partially restores downregulation of *Brca1* and *Brca2* mRNA.** NIH3T3-*Lin37^-/-^* cells were transfected with Lin37-WT-pRTS (WT) or Luci-pRTS (ctrl. construct). Cells were serum-starved (0 h) and restimulated to progress through the cell cycle (Mages et al. 2017). *Brca1* and *Brca2* mRNA levels were measured by RT-qPCR and normalized to values from WT transfections at 27 h. Two technical replicates, mean ± SD.
