# Supplementary material for: BRCA1 and BRCA2 gene expression: p53- and cell cycle-dependent repression requires RB and DREAM
Source: Cell Death Differ. 2025 Aug 22;33(1):51–63. doi: 10.1038/s41418-025-01566-9 (PMC12811384; doi:10.1038/s41418-025-01566-9)
Supplement: Supplementary file 6 — Legend Suppl. Fig. S3 [file 41418_2025_1566_MOESM6_ESM.docx]

**Legend Suppl. Fig. S3**

**A-MYB and B-MYB do not participate in activating *BRCA1* and *BRCA2* expression.** HCT116 cells were transfected with unspecific siRNA (Ctrl siRNA) and siRNA against A-MYB and B-MYB. Two days after knockdown transfection RNA was isolated and mRNA expression levels were determined by qPCR (Mean ± SD, n≥3, two-way ANOVA; ns, not significant; *p ≤ 0.05; **p ≤ 0.01; ***p ≤ 0.001).
