# Supplementary material for: BRCA1 and BRCA2 gene expression: p53- and cell cycle-dependent repression requires RB and DREAM
Source: Cell Death Differ. 2025 Aug 22;33(1):51–63. doi: 10.1038/s41418-025-01566-9 (PMC12811384; doi:10.1038/s41418-025-01566-9)
Supplement: Supplementary file 8 — Legend Suppl. Fig. S4 [file 41418_2025_1566_MOESM8_ESM.docx]

**Legend Suppl. Fig. S4**

**ChIP-seq data indicate RB and DREAM binding to the *BRCA1* and *BRCA2* promoters *in vivo*.** DNA segments of 4,000 base pairs surrounding the transcription start site of the human **(A)** *BRCA1*, **(B)** *BRCA2*, **(C)** *ORC1*, **(D)** *CCNB2*, and **(E)** *GAPDH* genes (genome version hg38) are displayed with a layered H3K27Ac track of seven cell lines from ENCODE and the density track of the ReMap Atlas using the UCSC Genome Browser. These tracks were compared to peak-of-peaks analyses of p53, DREAM, FOXM1-MMB, and RB from the targetgenereg.org database. The analysis for *GAPDH* served as a negative control.
