# Supplementary material for: BRCA1 and BRCA2 gene expression: p53- and cell cycle-dependent repression requires RB and DREAM
Source: Cell Death Differ. 2025 Aug 22;33(1):51–63. doi: 10.1038/s41418-025-01566-9 (PMC12811384; doi:10.1038/s41418-025-01566-9)

Figure 1A

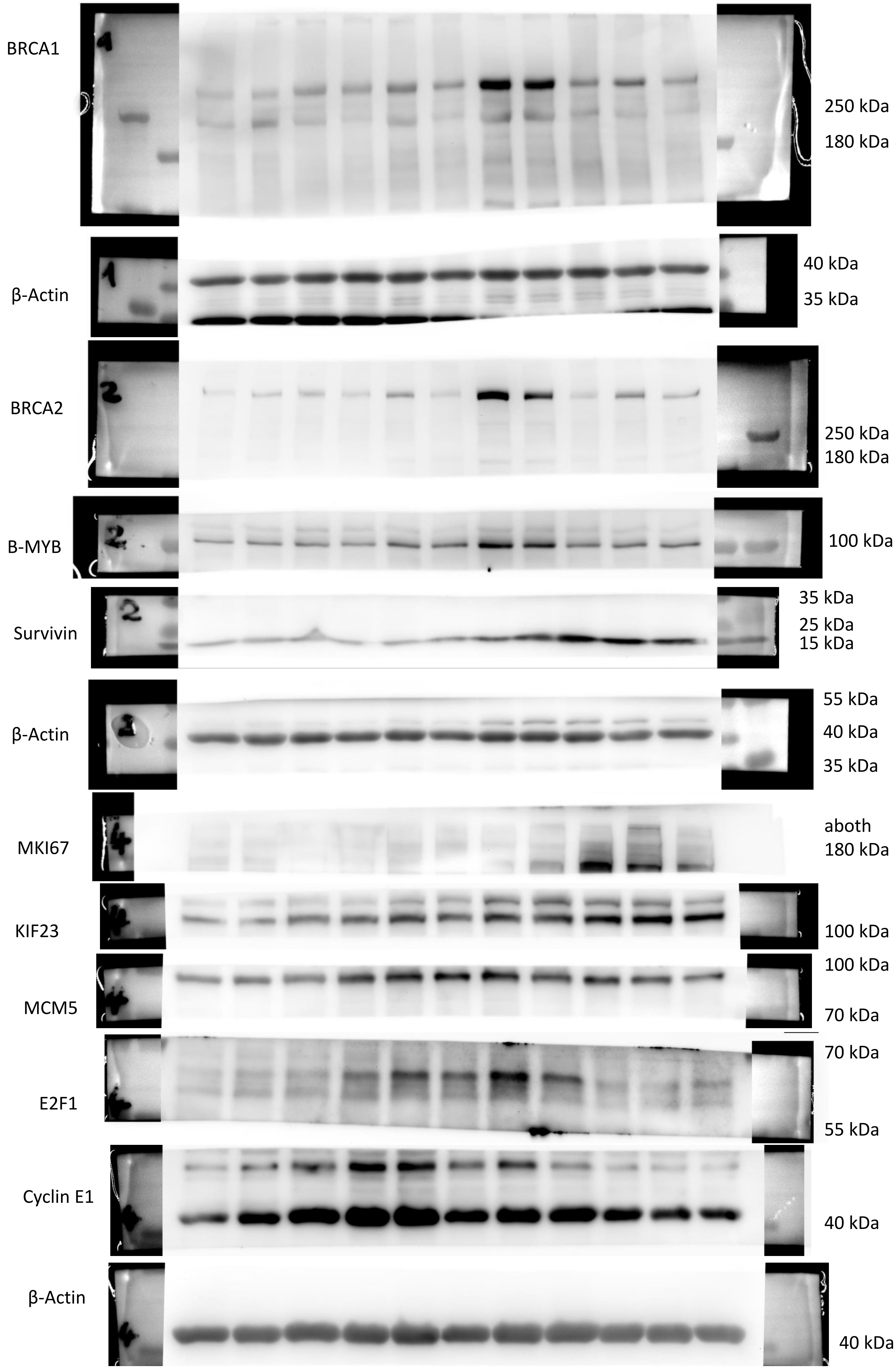
BRCA1
250 kDa
180 kDa
β-Actin
40 kDa
35 kDa
BRCA2
250 kDa
180 kDa
B-MYB
100 kDa
Survivin
35 kDa
25 kDa
15 kDa
β-Actin
55 kDa
40 kDa
35 kDa
MKI67
aboth
180 kDa
KIF23
100 kDa
MCM5
100 kDa
70 kDa
E2F1
70 kDa
55 kDa
Cyclin E1
40 kDa
β-Actin
40 kDa

Figure 4C – density arrested

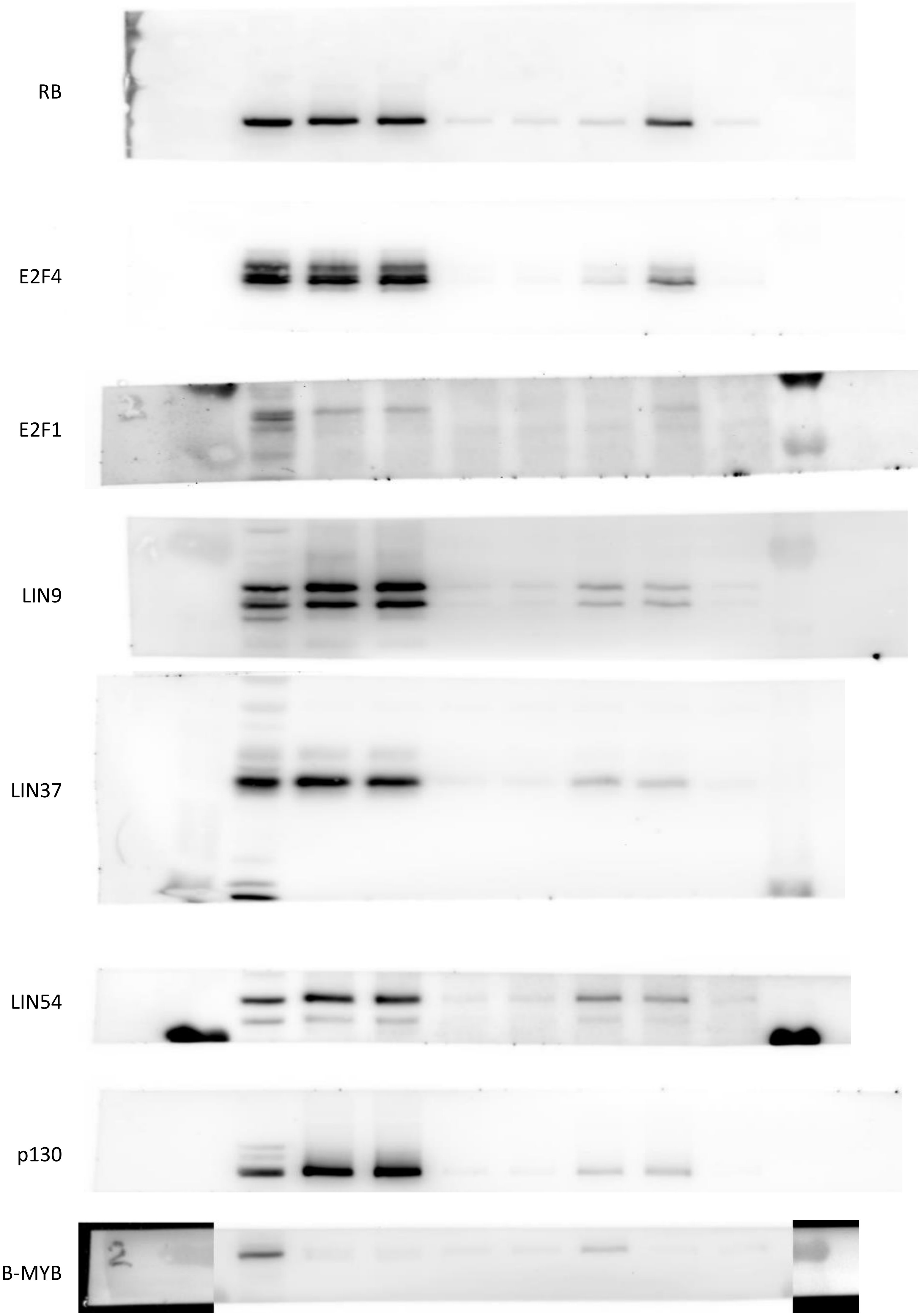

Figure 4C – restimulated

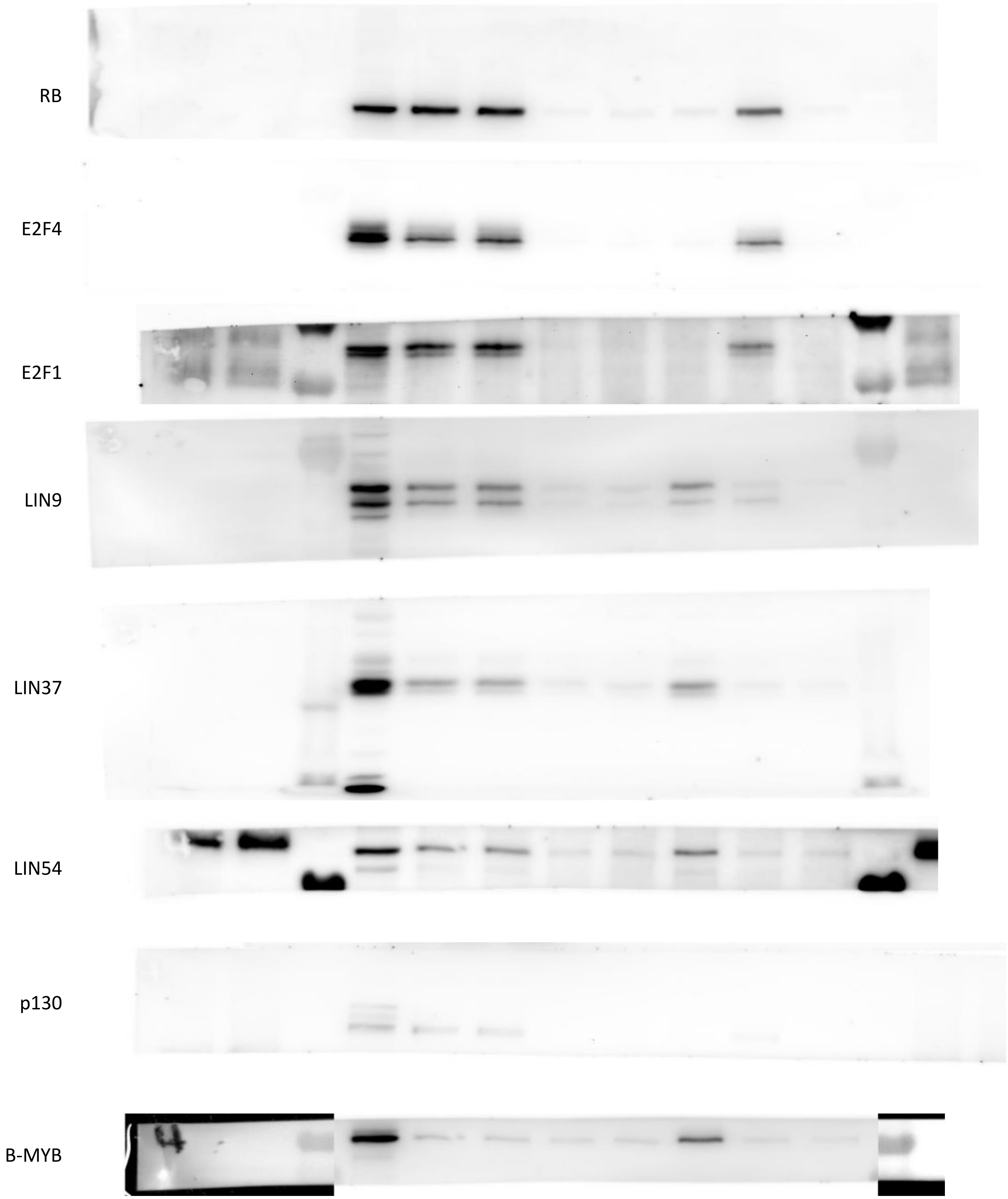

Figure 4D

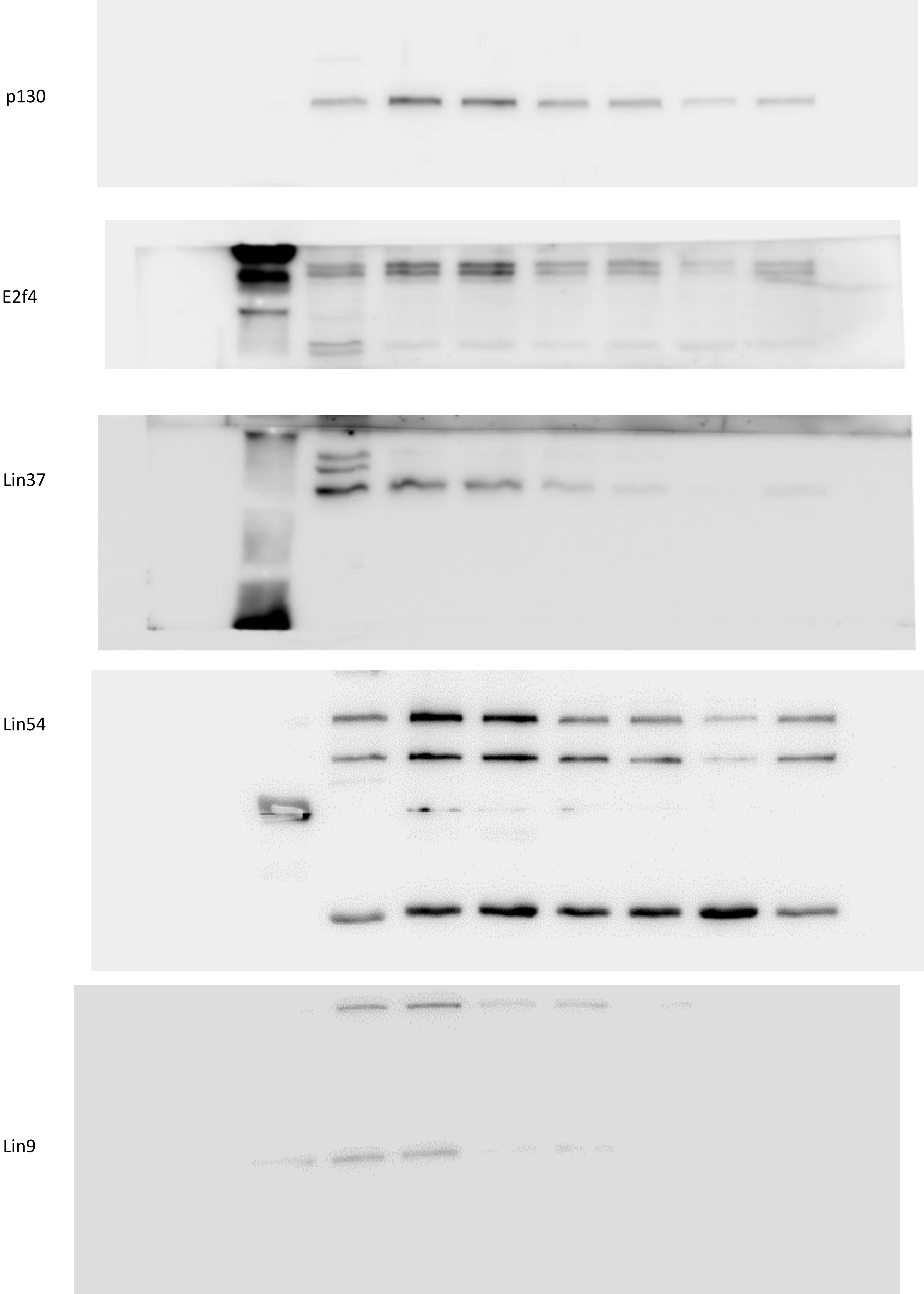

Figure 4E

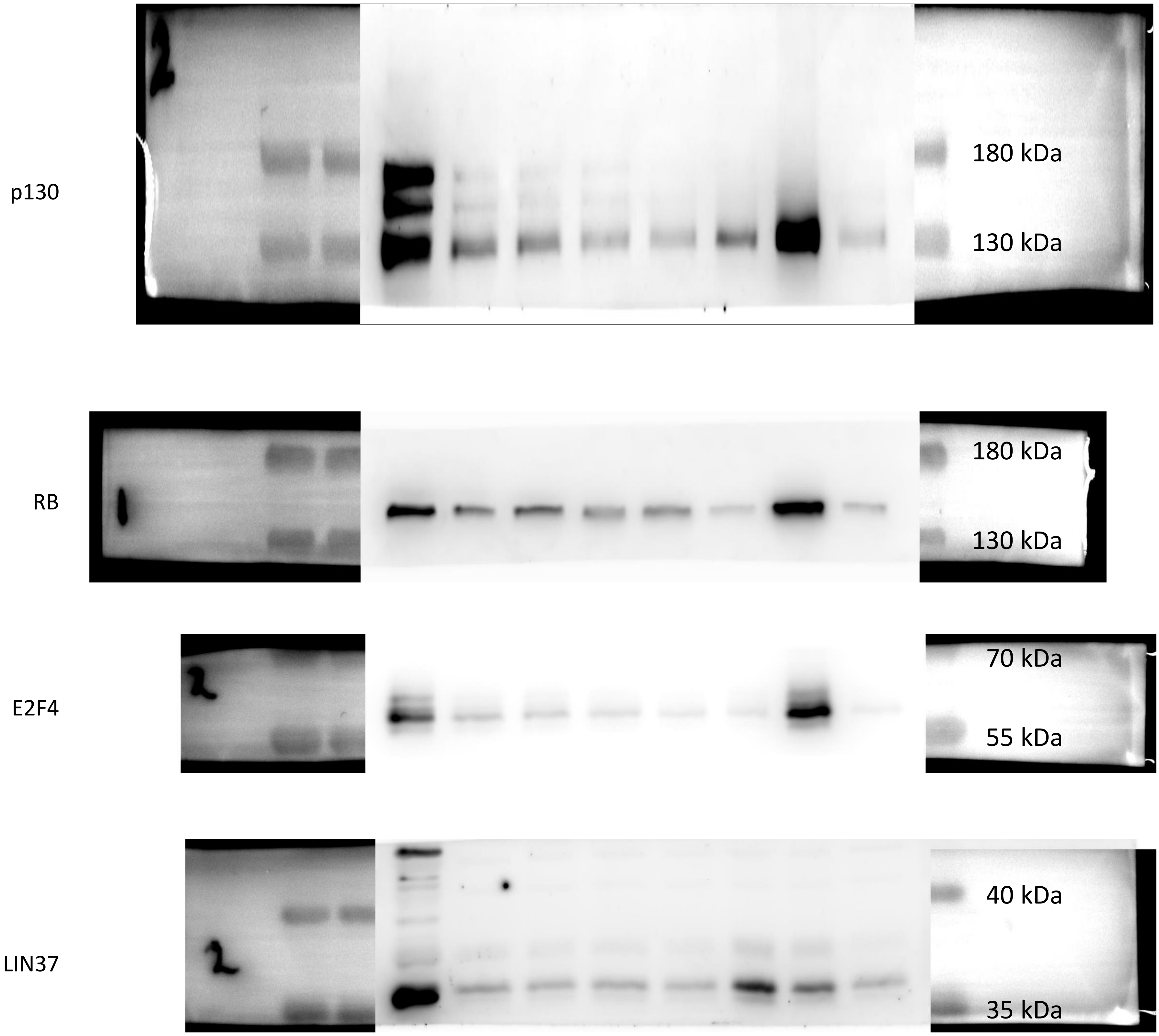
p130
180 kDa
130 kDa
RB
180 kDa
130 kDa
E2F4
70 kDa
55 kDa
LIN37
40 kDa
35 kDa

Figure S4

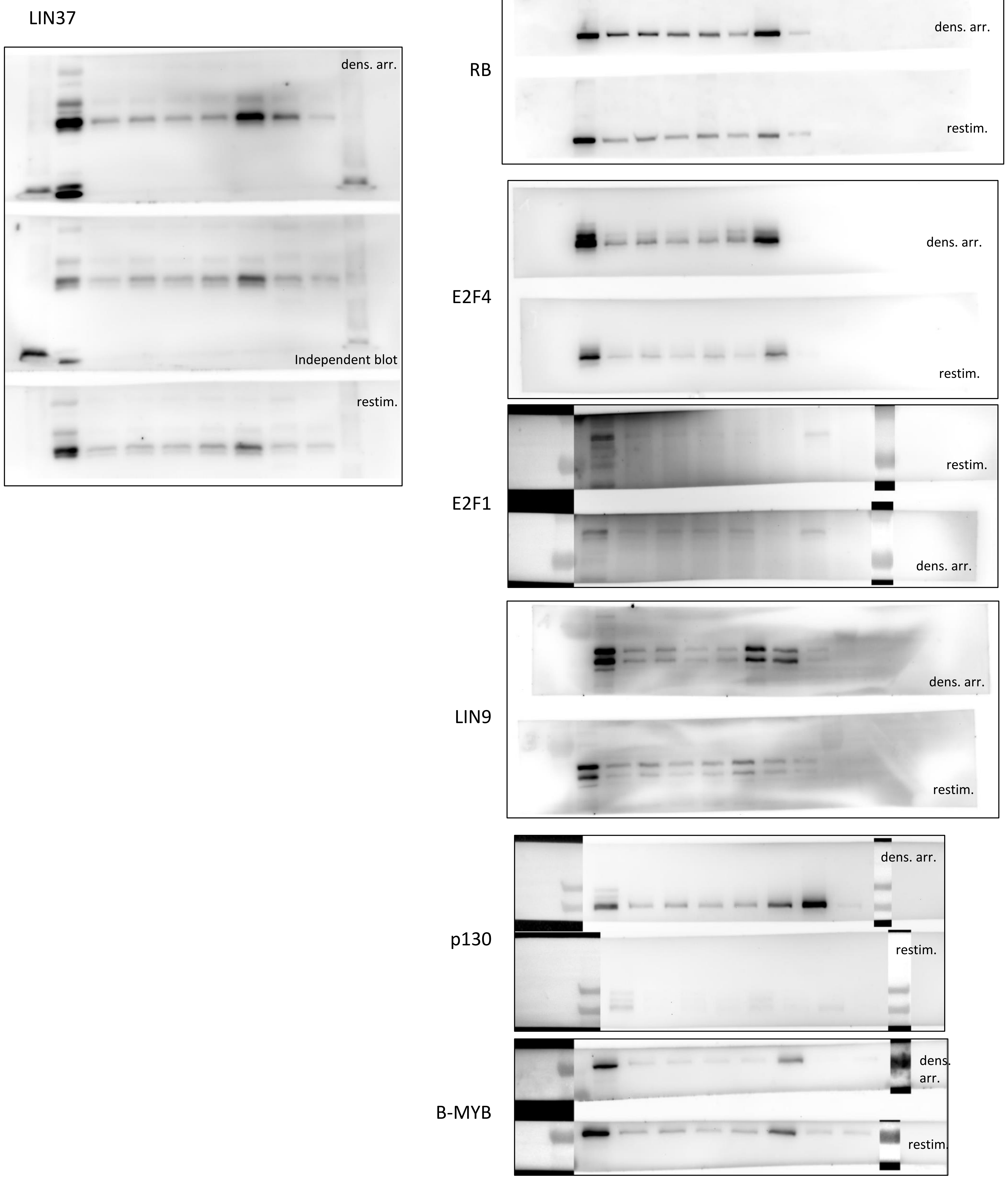

LIN37
dens. arr.
Independent blot
restim.
RB
dens. arr.
restim.
E2F4
dens. arr.
restim.
E2F1
restim.
dens. arr.
LIN9
dens. arr.
restim.
p130
dens. arr.
restim.
B-MYB
dens. arr.
restim.

Figure 6C

BRCA1

BRCA2

LIN37

RB

p21

β-actin

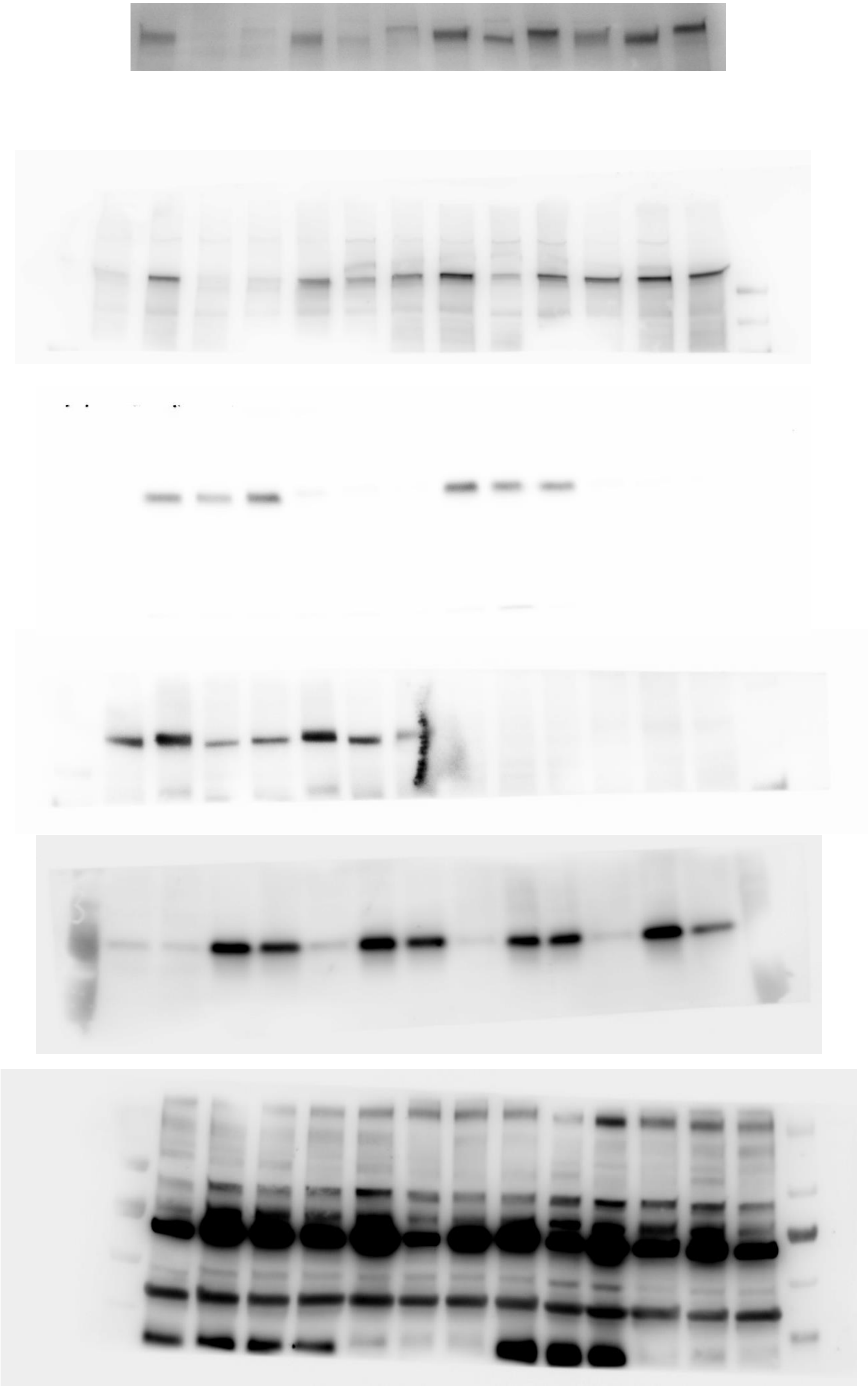

Supplement: Supplementary file 9 — Suppl. Fig. Uncropped Westerns [file 41418_2025_1566_MOESM9_ESM.pdf]
